# SequencErr: measuring and suppressing sequencer errors in next-generation sequencing data

**DOI:** 10.1186/s13059-020-02254-2

**Published:** 2021-01-25

**Authors:** Eric M. Davis, Yu Sun, Yanling Liu, Pandurang Kolekar, Ying Shao, Karol Szlachta, Heather L. Mulder, Dongren Ren, Stephen V. Rice, Zhaoming Wang, Joy Nakitandwe, Alexander M. Gout, Bridget Shaner, Salina Hall, Leslie L. Robison, Stanley Pounds, Jeffery M. Klco, John Easton, Xiaotu Ma

**Affiliations:** 1 Department of Computational Biology, St. Jude Children’s Research Hospital, Memphis, TN USA; 2 Department of Computer Science, University of Memphis, Memphis, TN USA; 3 Memphis, USA; 4 Department of Epidemiology & Cancer Control, St. Jude Children’s Research Hospital, Memphis, TN USA; 5 Department of Pathology, St. Jude Children’s Research Hospital, Memphis, TN USA; 6 Discovery Life Sciences, Huntsville, AL USA; 7 Department of Biostatistics, St. Jude Children’s Research Hospital, Memphis, TN USA

**Keywords:** Sequencer/instrument error, Error suppression, DNA sequencing

## Abstract

**Background:**

There is currently no method to precisely measure the errors that occur in the sequencing instrument/sequencer, which is critical for next-generation sequencing applications aimed at discovering the genetic makeup of heterogeneous cellular populations.

**Results:**

We propose a novel computational method, SequencErr, to address this challenge by measuring the base correspondence between overlapping regions in forward and reverse reads. An analysis of 3777 public datasets from 75 research institutions in 18 countries revealed the sequencer error rate to be ~ 10 per million (pm) and 1.4% of sequencers and 2.7% of flow cells have error rates > 100 pm. At the flow cell level, error rates are elevated in the bottom surfaces and > 90% of HiSeq and NovaSeq flow cells have at least one outlier error-prone tile. By sequencing a common DNA library on different sequencers, we demonstrate that sequencers with high error rates have reduced overall sequencing accuracy, and removal of outlier error-prone tiles improves sequencing accuracy. We demonstrate that SequencErr can reveal novel insights relative to the popular quality control method FastQC and achieve a 10-fold lower error rate than popular error correction methods including Lighter and Musket.

**Conclusions:**

Our study reveals novel insights into the nature of DNA sequencing errors incurred on DNA sequencers. Our method can be used to assess, calibrate, and monitor sequencer accuracy, and to computationally suppress sequencer errors in existing datasets.

## Introduction

**Fig. 1:**
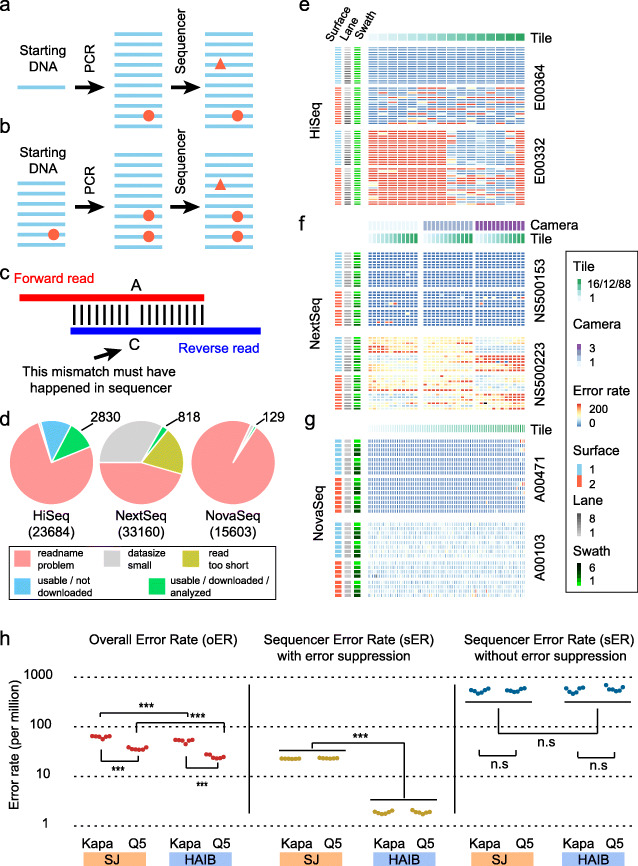
Measuring sequencer error rates. **a**, **b** Reference DNA method, where large amounts of reference DNA are needed. This can be achieved by starting from **a** small amounts of DNA/cells (to minimize inter-molecule/cell genetic heterogeneity) followed a by a large number of PCR cycles and sequencing. Alternatively, we can start from **b** large amounts of starting DNA/cells followed by a small number of PCR cycles (to minimize PCR errors) and sequencing. In both approaches, mutations/PCR errors (red dots) before sequencing can confound the sequencer error rate estimate (red triangles). **c** We interrogate the sequencer errors by focusing on discordant bases between forward and reverse reads of the same DNA segment within the overlapping regions. Such mismatches must have happened in the sequencer. **d** Public datasets produced by HiSeq, NextSeq, and NovaSeq as of December 2019. Datasets without proper read names, with very small sizes, or with very short reads (so that overlap is minimal) are not suitable for our analysis (see the “Methods” section). HiSeq has the most suitable datasets and we downloaded and analyzed ~ 50% of these. **e**–**g** Tile-level error rate across representative sequencers for **e** HiSeq, **f** NextSeq, and **g** NovaSeq. In each panel, a “good” sequencer (top) is illustrated with a “problematic” sequencer (bottom), where sequencer identifiers are indicated on the right. **h** Comparison of overall error rate (oER) and sequencer error rate (with or without computational error suppression) measurements on a common DNA library (generated by PCR enzymes Kapa and Q5) sequenced by two sequencing providers (St. Jude Children’s Research Hospital Computational Biology Genomics Laboratory (SJ) and HudsonAlpha Institute of Biotechnology (HAIB)), with two different NovaSeq sequencers. Tile arrangements are determined according to vendor documentation (see the “Methods” section). Tile-level error rates are capped at 200 per million for visualization purposes. ***Significant Wilcoxon rank-sum test (two-sided) *P* value (< 0.01). n.s, not significant (*P* > 0.01)

The sensitive detection of rare genetic variants in a population of cells is critical for multiple applications in biology and medicine, including industrial microbial engineering [1], drug-resistance management in infectious disease [2], and in oncology for the early detection [3] or non-invasive diagnosis [4] (known as liquid biopsy) of cancers. In these scenarios, it is highly desirable to detect bona fide mutations present at minuscule frequencies. Deep DNA sequencing by next-generation sequencing (NGS) technology holds great promise, but the sequencing accuracy remains a bottleneck for these applications. For example, the overall DNA sequencing error rate was reported to be > 1000 per million (pm) from 2011 to 2018 [2, 5–7]. We recently discovered that the overall NGS error rate can be computationally suppressed nearly 100-fold to between 10 pm and 100 pm through modeling of alignment artifacts and quality variation [8]. This in turn enabled a series of applications requiring highly sensitive detection of low-frequency events [9, 10]. In these reports, overall error rate (oER) is measured by a “reference DNA” method [8] where the DNA library is assumed to be mutation-free. However, generating mutation-free DNA is itself a challenge. For example, the highest-fidelity polymerase Q5 was reported to have an error rate (pER, for PCR error rate) of 0.53 pm [11], which can lead to genetically heterogeneous DNA molecules upon PCR amplification (Fig. 1a). In addition, human cells are estimated to have a mutation rate of ~ 10^−8^ (0.01 pm) per position per haploid genome [12, 13]. As a result, DNA extracted from cell populations can have bona fide low-frequency mutations (Fig. 1b). Therefore, in reference DNA-based methods, the sequencing readout is a product of mutations in cells or misincorporations during PCR amplification, as well as errors induced in sequencers (Fig. 1a, b). As a result, it remains unknown how to precisely measure sequencer error rates (sER), which are necessary to make informed decisions about platform (e.g., HiSeq vs NovaSeq) and sequencer (i.e., actual instrument) choices for deep sequencing applications, to diagnose sequencer problems, and to improve the accuracy of DNA sequencing.

In this work, we present a novel computational method, SequencErr, to precisely measure sER. The key idea is to utilize the paired-end sequencing methodology (Fig. 1c), which was designed to double the sequencing yield by sequencing the input DNA molecule from both ends. When the input DNA molecule is short, forward and reverse reads overlap and the overlapping base pairs are sequenced twice. Identical readouts are expected if there are no sequencer errors, and discordance between forward and reverse reads must be a result of an error in the sequencer (Fig. 1c). We note that overlapping reads have been extensively utilized to reduce errors in the literature [14–18], and the novelty here is to use overlapping reads to investigate the accuracy of the sequencer, flow cells, etc. We investigated error patterns associated with platforms, sequencers, flow cells, and tiles in flow cells (see Additional file 1: Supplementary Note 1 for cartoon illustration) by using 3777 datasets from 75 research institutions in 18 countries (Additional file 2: Table S1; see the “Methods” section). Our results provide critical insights into sequencer accuracy and suggest future directions to enhance instrument accuracy.

### Calculating sequencer error rate with SequencErr

For a given read pair *r*, we denote the number of overlapping base pairs between forward and reverse reads as *n*_*r*_. The number of sequenced bases in this region is 2*n*_*r*_, where *r* = 1, …, *K*, and *K* is the number of read pairs in each evaluation unit (e.g., one tile). We denote the number of base pairs with a mismatch between forward and reverse reads as *m*_*r*_. Considering the nested structure of reaction units in a sequencing run, where a read belongs to a tile, a tile belongs to a swath, a swath belongs to a lane, a lane belongs to a surface, and a surface belongs to a flow cell (Additional file 1: Supplementary Note 1), we can define the sequencer error rate at different granularity scales. For example, the tile-level sER can be calculated as:

1 $$ {e}_t=\frac{\sum_r{m}_r}{\sum_r2{n}_r}\ \mathrm{where}\ \mathrm{read}\ \mathrm{pair}\ r\in \mathrm{tile}\ t $$

Similarly, the flow cell-level sER is defined as:

2 $$ {e}_f=\frac{\sum_r{m}_r}{\sum_r2{n}_r}\ \mathrm{where}\ \mathrm{read}\ \mathrm{pair}\ r\in \mathrm{flow}\ \mathrm{cell}\ f $$

and the surface-level sER is defined as:

3 $$ {e}_s=\frac{\sum_r{m}_r}{\sum_r2{n}_r}\ \mathrm{where}\ \mathrm{read}\ \mathrm{pair}\ r\in \mathrm{surface}\ s $$

Physical location information of reads, such as sequencer and flow cell identifiers and tile numbers, is stored in the read name, which is critical for our analysis (see the “Methods” section). We do not specifically analyze the lane effect because it is custom configurable (see the “Methods” section; Additional file 1: Fig. S1).

### Measuring overall sequencing error rate at base pair level

When a reference DNA library has been deeply sequenced (e.g., with > 1,000,000× depth), the known wild-type bases can be used to calculate overall error rate (oER) in a site-specific fashion [8] as follows:

4 $$ {\mathrm{error}\ \mathrm{rate}}_i\left(g>m\right)=\frac{\#\mathrm{reads}\ \mathrm{with}\ \mathrm{nucleotide}\ m\ \mathrm{at}\ \mathrm{position}\ i}{\mathrm{Total}\#\mathrm{reads}\ \mathrm{at}\ \mathrm{position}\ i} $$

where *g* indicates the reference allele at genomic locus *i*, and *m* represents each of the three possible substitutions caused by sequencing error. For example, at a given site with reference allele A, we can calculate oER for the three possible mismatches, A>C, A>G, and A>T. Note that oER is a product of bona fide cellular mutations, PCR errors (pER), and sequencer error (sER). It is different from the sER measured in Eqs. 1, 2, and 3. The oER can be used to compare datasets generated by different sequencers, or to compare datasets with or without removing outlier tiles as discussed later.

### Datasets for benchmarking SequencErr

Many datasets across a broad spectrum of platforms, sequencers, flow cells, and samples are needed to test the efficacy of our method. For this purpose, we analyzed datasets from the public repository NCBI Sequence Read Archive (SRA) (see the “Methods” section) for three major platforms—HiSeq, NextSeq, and NovaSeq (Fig. 1d, Additional files 3, 4, 5: Tables S2, S3, S4). HiSeq is the most common of these due to its earlier release (2010), followed by NextSeq (2014) and NovaSeq (2017). A significant challenge is that read names in many datasets have been reformatted in NCBI SRA (see the “Methods” section, Additional file 1: Supplementary Notes 2-3, Additional files 3, 4, 5: Tables S2, S3, S4), possibly to save storage space. This resulted in only 5.2% of the public datasets (*n* = 72,447) being suitable for our study (Fig. 1d). These were generated in 75 research institutes across 18 countries (Additional file 2: Table S1).

**Fig. 2:**
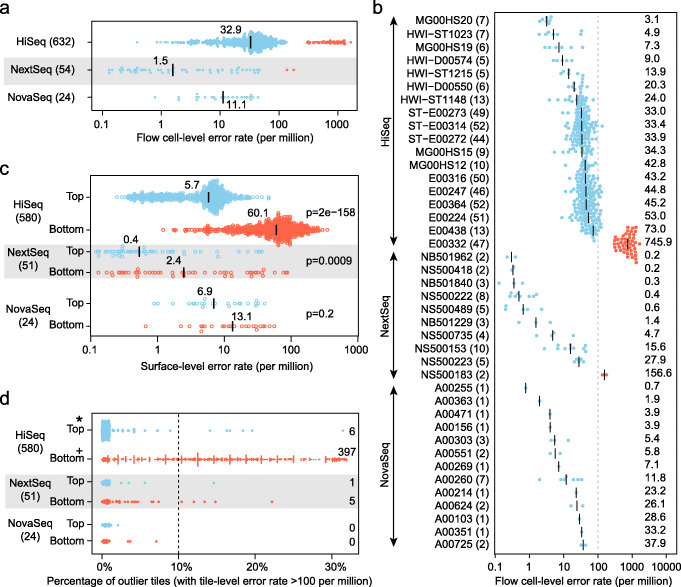
Analysis of sequencer errors. **a** Flow cell-level error rate distribution of common sequencing platforms including HiSeq, NextSeq, and NovaSeq (with the number of flow cells indicated). Outlier flow cells are highlighted in red. Vertical bars indicate the medians. **b** Flow cell-level error rate across sequencers (instrument identifiers and number of flow cells in parentheses; due to limited availability, sequencers with at least 5, 2, and 1 flow cells are included for HiSeq, NextSeq, and NovaSeq, respectively; see full data in Additional file 1: Fig. S2). Medians are indicated with a vertical black bar and on the right margin of the figure. Red dots indicate outlier sequencers defined at error rate cutoff 100 per million (10^−4^; vertical dashed line). **c** Comparison of error rate between the top and bottom surfaces of flow cells. Medians are indicated by vertical bars. Two-sided Wilcoxon rank-sum test *P* values are indicated for each platform in the right margin. **d** Prevalence of outlier tiles across flow cells, stratified by the top and bottom surfaces. Shown are percentages (*x*-axis) of outlier tiles with a high error rate (> 100 per million) for top (blue) and bottom (red) surfaces of each flow cell across three platforms (*y*-axis). For each platform, the number of flow cells with more than 10% (dashed vertical line) high error rate tiles is indicated by the numbers on the right. *For HiSeq top surfaces, percentages < 1% are replaced with a random number in [0%, 1%] for display purposes. ^+^For HiSeq bottom surfaces, percentage > 30% are replaced with a random number in [30%, 32%] for display purposes

We analyzed the datasets according to flow cells considering that multiple samples (i.e., multiplexing with barcode) can be pooled in one flow cell, and a sample may be sequenced in multiple flow cells. In fact, different samples pooled in the same flow cell tend to have similar tile-level error rates, indicating a minimal sample effect (Additional file 1: Fig. S1; Additional file 6: Table S5). This resulted in 632, 54, and 24 flow cells from 108 HiSeq, 20 NextSeq, and 13 NovaSeq sequencers, respectively (Fig. 2a, Additional file 7: Table S6). Tile-level error rates (Eq. 1) of representative flow cells are illustrated in Fig. 1e–g, where the variability of error rates among sequencers, flow cells, surfaces, and tiles can be observed.

### Comparison of overall error rate and sequencer error rate measures

As illustrated in Fig. 1a–c, the overall error rate (oER, Eq. 4) is a measure of sequencing errors with a mixture of error sources, including PCR artifacts and sequencer artifacts. On the other hand, the sequencer error rate (sER, Eqs. 1–3) is a direct measure of errors specific to a sequencer. We, therefore, compared these measures by using a previously published amplicon sequencing dataset (ENA project ID PRJEB35986) [8]. In this dataset, genomic loci flanking the spike-in somatic mutations are known to be wild-type and are used to measure the oER (Eq. 4). On the other hand, the sER was calculated with Eq. 2. Because exactly the same DNA library (generated by PCR enzyme Kapa and Q5, respectively) was sequenced by different NovaSeq sequencers from different sequencing providers (SJ: St. Jude Children’s Research Hospital; HAIB: HudsonAlpha Institute for Biotechnology) [8], it also provided a unique opportunity to benchmark the instruments. Consistent with the expectation that sER is a subset of the oER, the measured oER is consistently higher than sER (Fig. 1h). Strikingly, data generated by SJ demonstrated a significantly (two-sided Wilcoxon rank-sum test, *P* < 0.01) higher oER than that generated by HAIB, indicating a strong contribution of sequencer errors. Indeed, the sER of SJ is also significantly (two-sided Wilcoxon rank-sum test, *P* < 0.01) higher than that of HAIB. The consistent significantly (two-sided Wilcoxon rank-sum test, *P* < 0.01) lower overall error rate of Q5 than that of Kapa is consistent with our previous findings [8]. This data supports the value of measuring sER because lower sequencer error rate can result in lower overall error rate and measuring sER might help choosing the best sequencers for deep sequencing applications. For example, NovaSeq and NextSeq are on average preferred over HiSeq sequencers. Because different sequencers can have dramatically different error rates (such as the two NovaSeq sequencers studied here), specific sequencers with lower error rates are preferred.

To understand the effect of computational error suppression in sER, we performed a similar analysis as above except without computational error suppression (i.e., no quality filtering on mapping quality and Phred scores). As a result (Fig. 1h), the sER is now close to 1000 pm (10^−3^), which is consistent with previous reports [2, 6]. This result reinforced our previous observation that computational error suppression can lead to 10- to 100-fold error rate reduction [8]. With this observation, we will apply computational error suppression to calculate sER hereafter unless otherwise stated.

### Comparison of sequencer error rates between platforms

We first studied the general sequencer error rate (sER) patterns associated with the HiSeq, NextSeq, and NovaSeq sequencing platforms. For this purpose, we summarized flow cell-level sER using Eq. 2. As can be seen from Fig. 2a and Additional file 7: Table S6, HiSeq, NovaSeq, and NextSeq have an average sER of 32.9, 11.1, and 1.5 pm, respectively. Because HiSeq and NovaSeq have the highest throughput, and possibly the most popular usage, we conclude that the current sER is ~ 10 pm.

We noticed that many flow cells in HiSeq and NextSeq platforms demonstrate elevated sER (red dots in Fig. 2a). To test if there are systematic error sources, we reorganized the data by focusing on sequencers that have data from multiple flow cells (Fig. 2b, Additional file 1: Fig. S2 and Additional file 7: Table S6). Only a few sequencers have sER greater than 100 pm where all flow cells appear to be affected (red dots in Fig. 2b and Additional file 1: Fig. S2). Therefore, we define outliers by using 100 pm as threshold hereafter. Interestingly, tiles in physical proximity in outlier sequencers tend to have concordant error rate patterns between flow cells (Additional file 1: Fig. S3, Additional file 8: Table S7), indicating a sequencer problem. On the other hand, flow cell-level sER appears to be highly stable across runs within the non-outlier sequencers (Fig. 2b, Additional file 1: Fig. S2), indicating that a successful initial sequencing experiment may ensure the generation of high-quality data across many flow cells. We identified two sequencers (E00332 and NS500183, 1.4% of 141 sequencers; Fig. 2b, Additional file 7: Table S6) as outlier sequencers and corresponding datasets were omitted from further analyses. In the non-outlier sequencers (Additional file 7: Table S6), 17 flow cells (2.5%, *n* = 661) have marginally high error rate (between 100 pm and 150 pm). One (0.15%, *n* = 661) flow cell (C5E39ANXX, Additional file 7: Table S6) has a very high error rate (15,225 pm) and was omitted from further analyses.

### Flow cell surfaces

Because it appears that the top surface has a lower sER than the bottom surface in the representative flow cells (Fig. 1e–g), we next calculated sER in the top and bottom surfaces (Eq. 3) for each flow cell. As can be seen in Fig. 2c and Additional file 9: Table S8, the top surfaces have significantly (two-sided Wilcoxon rank-sum test, *P* < 0.01) lower median sER than bottom surfaces for HiSeq and NextSeq. For NovaSeq, the top surface tends to have lower median sER than the bottom surface, although statistical significance is not reached. This data indicates a systematic problem in the bottom surfaces of flow cells and an apparent quality improvement of bottom surfaces in the newer sequencers.

### Flow cell tiles

Because there are outlier tiles with dramatically elevated sER at the flow cell level (Fig. 1e–g), we next studied the extent of outlier tiles. We defined a tile as an outlier if its sER (Eq. 1) is > 100 pm, with the observation that 96.3% flow cells have sER < 100 pm (Fig. 2b, Additional file 1: Fig. S2, Additional file 7: Table S6). As can be seen in Fig. 2d and Additional file 1: Fig. S4 and Additional file 10: Table S9, 6 out of 580 (1%) HiSeq flow cells have more than 10% outlier tiles in the top surface, while 397 out of 580 (68%) HiSeq flow cells have more than 10% outlier tiles in the bottom surface. For NextSeq, 1 out of 51 (2%) flow cells in the top surface and 5 out of 51 (10%) flow cells in the bottom surface have more than 10% outlier tiles. None of the 24 NovaSeq flow cells have more than 10% outlier tiles. This data indicates that a high number of HiSeq flow cells have quality problems originating from the bottom surface. An improvement of bottom surface quality from HiSeq (68% flow cells) to NextSeq (10% flow cells) and NovaSeq (0% flow cells) is observed (Fig. 2c). Notably, 44.4% and 88.9% of NovaSeq flow cells have at least one outlier tile in the top and bottom surface (*n* = 18; Additional file 1: Fig. S4, Additional file 11: Table S10). Overall, 94.2% (*n* = 580), 45% (*n* = 51), and 95.8% (*n* = 24) of HiSeq, NextSeq, and NovaSeq flow cells have at least one outlier tile, respectively (Additional file 9: Table S8).

We next asked if the outlier error-prone tiles in non-outlier sequencers demonstrate patterns in physical locations. As it turns out, the outlier tiles have a roughly uniform distribution of physical positions (Additional file 1: Fig. S5; Additional file 12: Table S11), although the enrichment of outlier tiles with higher position number is observed in HiSeq top surfaces while enrichment of outlier tiles with lower position number is observed in NovaSeq bottom surfaces. It should be noted that the current total number of publicly accessible datasets for NextSeq and NovaSeq is quite limited, and more robust estimates can be achieved when more datasets are evaluated.

### Effect of sequencer on the overall sequencing error rate

**Fig. 3:**
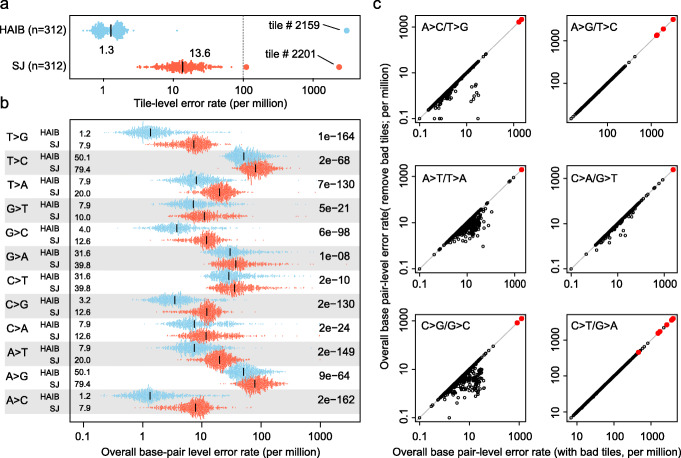
Effect of sequencer error suppression on overall sequencing error rates. **a** A NovaSeq sequencer from HudsonAlpha Institute of Biotechnology (HAIB) and a NovaSeq sequencer from St. Jude Children’s Research Hospital (SJ) have dramatically different tile-level error rates estimated by using reference DNA library COLO829 with 1:1000 dilution (see the “Methods” section). Tiles with an error rate > 100 per million (vertical dashed line) are defined as outlier tiles (large solid dots). **b** Overall sequencing error rate from these two sequencers on reference DNA library COLO829. Median error rates (vertical black bars) of each misincorporation type on known wild-type bases (see the “Methods” section) are indicated in the left margin of the panel, and the two-sided Wilcoxon rank-sum test *P* values between HAIB and SJ for each misincorporation types are indicated in the right margin of the panel. **c** Effect of removing outlier tiles in the HAIB dataset (1:1000 dilution, see the “Methods” section) defined in **a**. Each dot represents the site-specific error rate of given misincorporation types with (*x*-axis) and without (*y*-axis) the outlier tiles. Red dots: spike-in true mutations (see the “Methods” section). Diagonal (no change) is indicated by gray lines

We next studied the effect of different sequencers on oER (Eq. 4) in more detail. For this purpose, we utilized the COLO829 dilution datasets (see the “Methods” section) published previously [8], where a common reference DNA library was sequenced by two sequencing centers, HAIB (HudsonAlpha Institute of Biotechnology, Huntsville, AL) and SJ (St. Jude Children’s Research Hospital, Memphis, TN) using two NovaSeq sequencers, A00363 (HAIB) and A00214 (SJ). As can be seen in Fig. 3a and Additional file 13: Table S12, these sequencers have a 10-fold error rate difference, by which we expect the dataset generated by HAIB to have a lower oER. Notably, the HAIB dataset has a bi-modal distribution of tile-level error rates. In fact, the lower error rate tiles are located on the top surface and the higher error rate tiles are located on the bottom surface (Additional file 1: Fig. S6a, Additional file 13: Table S12), reinforcing the observation of elevated error rates on bottom surfaces (Fig. 2c).

We calculated the site-specific overall sequencing error rate (oER) as previously described (Eq. 4; Additional file 14: Table S13). As can be seen in Fig. 3b, the oER of HAIB is between 4- and 7-fold lower than that of SJ for A>C/T>G and C>G/G>C misincorporations, and the oER difference is less prominent for other error types. This result indicates two possibilities: (a) the sequencer might have elevated misincorporation types, such as A>C/T>G, or (b) the reduction of sER has a negligible effect because PCR error rate (pER) is an order of magnitude greater than sER. To test these hypotheses, we compared the sER (Eq. 2) against oER (Eq. 4). As it turned out, we found a statistically significant negative correlation between sER and oER in datasets from both A00214 and A00363 (Additional file 1: Fig. S6c-d), indicating dramatically different misincorporation types between NovaSeq sequencer and PCR enzymes.

### Removing outlier poor-quality tiles improves overall sequencing error rate

Because there appear to be extreme outlier tiles in HAIB sequencer A00363 (tile #2159) and SJ sequencer A00214 (tile # 2201; Fig. 3a), we next studied the effect of removing outlier tiles on the overall sequencing error rate (oER). For this purpose, we compared site-specific oER (Eq. 4; Additional file 14: Table S13) by excluding outlier tiles. As can be seen in Fig. 3c and Additional file 1: Fig. S7a and S8a, 7.6% of C>G/G>C errors have > 2-fold error rate reduction, with a maximum reduction of 24-fold, followed by 6% of A>T/T>A errors with > 2-fold error rate reduction in the HAIB dataset. On the other hand, the allele fractions of spike-in true mutations (red dots, Fig. 3c, see the “Methods” section) are not affected by the removal of outlier tiles. Therefore, we conclude that the removal of outlier error-prone tiles can further reduce the overall error rate. Notably, the numeric error suppression in A00214 appears to have a much less dramatic effect than in A00363 (which has lower sER; Additional file 1: Fig. S7 and S8), indicating that removal of outlier tiles is most effective when the instrument/flow cell is of higher accuracy.

### Comparison of SequencErr with FastQC

Because SequencErr is designed to understand sequencing quality, which is conceptually similar to FastQC [19]—a frequently used quality control tool for sequencing datasets—we also benchmarked our method against FastQC. However, these two methods are fundamentally different in their design principles. FastQC operates on Phred scores of sequenced bases (regardless of the actual sequence identity) and measures a sample-level quality metric as the percent of bases with the Phred score above a given threshold, e.g., Q30 percentage. In contrast to FastQC, SequencErr compares the actual sequence identity of aligned forward and reverse reads, although quality filters including Phred score distribution are applied to remove poor-quality data (see the “Methods” section). We used the previously published dataset [8] for this comparison. As seen in Additional file 1: Fig. S9a, the FastQC evaluation shows that the Q30 percentage is > 98% across all sequencing cycles, which would be considered good quality. However, by using our Eq. 1 at the sequencing cycle level, we observed a stable error rate of ~ 11 per million across the sequencing cycles (Additional file 1: Fig. S9b). Strikingly, we were able to observe the effect of problematic tiles on the sequencing cycles (Additional file 1: Fig. S9b,c,f), which cannot be observed by reviewing FastQC output file (Additional file 1: Fig. S9a,e).

### Effect of DNA sequencing features on sequencer error rate

We next studied the effect of DNA sequence features on sequencer error rates, by focusing on (1) GC content, (2) read length, and (3) overall base quality, measured as a percentage of bases with Phred score greater than 30 (Q30 percentage). Because a large number of samples are needed to draw a robust conclusion, here we focused on 1167 publicly available HiSeq datasets (Additional file 3: Table S2). As seen from Additional file 1: Fig. S10, we did not detect a significant correlation between sER score and GC content and read length features, although a marginally significant negative correlation with Q30 percentage is observed, indicating that excessive percentage of low-quality bases can lead to unreliable sequencing output, even if after stringent quality filtering. This indicates that our SequencErr metric is being highly robust in measuring sequencer reliability in a wide range of parameter settings.

### Comparison of SequencErr with error correction methods

In addition to our error suppression method, which operates by identifying and filtering (i.e., suppression) unreliable reads, considerable efforts have been devoted to error correction methods, which operates by identifying DNA contexts (i.e., k-mer) that are error-prone and followed by modifying (i.e., correction) corresponding readout. To benchmark with these methods, we focused on two error correction methods, Lighter (v1.1.2) [20] and Musket (v1.1) [21], which are considered to have the top performance in a recent study by Mitchell et al. [22]. We focused the analysis by using two representative samples (ERR3781298 and ERR3790800, Additional file 15: Table S14) sequenced using SJ and HAIB sequencers from previously published dataset [8] where the mutant and wild-type sites are well-defined (Additional file 14: Table S13). Here, the raw FastQ files were run through Lighter and Musket to correct errors, followed by standard pileup (see the “Methods” section). As can be seen in Additional file 1: Fig. S11a-b, the overall sequencing error rate (oER) obtained by SequencErr far outperforms that of Lighter and Musket for all 12 possible nucleotide changes, although for C>T/G>A changes the difference is least dramatic. Interestingly, the overall error rate of Lighter and Musket does not appear to show improvement compared to the direct standard pileup. We next hypothesized that DNA sequence context-based modifications may lead to overcorrection, which could be reflected in overlapping forward-reverse reads which are expected to have perfect matches. An increase in the mismatch rate would indicate overcorrection. Indeed, we observed ~ 10-fold increased forward-reverse mismatches in this dataset by Lighter or Musket than no correction (SequencErr measurement) (Additional file 1: Fig. S11c-d). Taken together, we have demonstrated that our error suppression method outperforms error correction methods.

### Application of SequencErr on a non-human dataset

Although we have demonstrated SequencErr method can be used to measure sequencer fidelity in the above studies, it remains unknown whether it can be applied to non-human datasets. For this purpose, we applied SequencErr on a recently published *Severe acute respiratory syndrome coronavirus 2* (SARS-CoV-2) dataset (NCBI SRA BioProject PRJNA625551) to study the error rate of the corresponding sequencer. As can be seen in Additional file 1: Fig. S12, the involved flow cells/sequencer demonstrated a comparable sER with those observed in human datasets (red dashed line). We, therefore, conclude that our method can also be applied to non-human datasets, although we believe that a more extensive study on many different non-human datasets could significantly strengthen this conclusion, which is beyond the scope of this work.

## Conclusions

High-throughput DNA sequencing technology has found increasingly important applications in biology and medicine in the past decade, and sensitive detection of low-frequency mutations through ultra-high depth sequencing is of great interest in many aspects of biology and medicine, such as liquid biopsies and in the detection of minimal residual disease for different cancers after therapy. Precise identification of error sources in the many steps of DNA sequencing workflow, such as in sequencers, is the key to enhance DNA sequencing technologies for such applications. However, there is a lack of methods for measuring the fidelity of sequencers, partly due to the difficulty in deconvoluting PCR and sequencer errors. As a result, it is difficult to evaluate the performance of a given sequencer for applications requiring high sequencing fidelity.

In this work, we took advantage of the paired-end sequencing strategy to precisely evaluate the sequencer error rate (sER). We discovered that for HiSeq, NextSeq, and NovaSeq platforms, most sequencers have an error rate ~ 10 pm, though 1.4% of the sequencers in this study appear to be outliers with flow cells demonstrating sER > 100 pm. In addition to sER, our reported overall error rate (oER) of 10–100 pm (Fig. 1h), which includes other error sources such as PCR errors, is 10- to 100-fold lower than the generally reported accuracy of next-generation sequencing methods of 1000 pm (10^−3^) [2]. This reflects the successful “suppression” of a poor-quality subset of data by our novel strategy. On the other hand, this raised an interesting perspective on the base call quality scores, known as Phred score. Traditional Phred scores were calculated based on sequencing traces of A/C/G/T bases by summarizing it to features such as peak spacing, uncalled/called ratio, and peak resolution [23]. For example, the current NovaSeq platforms only report Phred to score up to 42, which corresponds to *P* value of ~ 10^−4^. Because our data indicated that the calls at many bases have an error rate close to 10^−5^ (at least in NovaSeq), we believe a re-evaluation of the current Phred score calculation is warranted by the instrument manufacturer. For example, it would be interesting to determine if the Phred score binning strategy that was intended for better data compression in NovaSeq data may lead to less accurate Phred score estimation.

Although our current method (and the previous method CleanDeepSeq [8]) is designed for ultra-deep sequencing applications where high accuracy (i.e., low error rate) is of pivotal importance, it can also be used to determine sequencer accuracy for standard sequencing projects such as whole-genome and whole-exome sequencing (such data can be used to help determine which instruments have the lowest error rate). This is because the typical tens to hundreds of sequencing depth of whole-genome and whole-exome sequencing are not powered to detect variants at 1% to 0.1% or even lower frequencies—which is, in fact, the aim of ultra-deep (tens of thousands or more) sequencing projects such as liquid biopsy [4]. In this ultra-deep sequencing scenario, the 30% data loss of our method, as reported previously [8], is not a significant concern as compared with the heavily redundant sequencing of the barcode-sequencing method—for example, the > 60,000× sequencing of a recent large-scale UMI method resulted in a 4577× UMI-collapsed net depth—a 13-fold redundancy [4]. It is possible that our method can be further refined to filter by reads/sequencing cycles as opposed to the current tile-based filtering, which is beyond the scope of this work.

Within non-outlier sequencers, the error rates of different flow cells appear to be rather stable, although there is 2.7% chance of observing outlier flow cells, indicating the need to continuously monitor the accuracy of sequencers. At the flow cell level, we found that it is common to observe a small fraction (usually less than 10% in NextSeq and NovaSeq, 68% in HiSeq bottom surface) of tiles with exceedingly high error rates. Overall, > 90% of HiSeq and NovaSeq flow cells have at least one outlier tile. We also discovered that the bottom surfaces of flow cells tend to have higher sequencer error rates. The bottom surface problem tends to be alleviated in NextSeq and NovaSeq compared to HiSeq sequencers. This data also indicates that sequencer, flow cell, and flow cell surface differences (in terms of error rate) persist even after initial error suppression.

By using a common DNA library (COLO829), we demonstrated that sequencers with higher sER can lead to an elevated oER, indicating the need for evaluating sequencers for applications that require high sequencing accuracy, such as ultra-high depth sequencing applications. By removing outlier tiles, we achieved the dramatic reduction of oER in some genomic loci, indicating the benefit of controlling quality at the tile level.

There are a few limitations in our study. First, we did not have as much data from NextSeq and NovaSeq sequencers compared to HiSeq sequencers. With the continuous production of large-scale datasets using NovaSeq, we expect to develop a more comprehensive picture of this platform in the future. Second, our method requires overlapping forward and reverse reads, which may be challenging for some applications such as whole-genome sequencing, where the insert size is generally large to maximize the sequencing yield. This might be overcome by mixing a small fraction of short DNA segments as an “internal standard” [24] to each whole-genome sequencing run to monitor the sequencer accuracy. Nevertheless, our ability to identify problematic flow cells by using whole-genome sequencing datasets (see the “Methods” section) on HiSeq sequencers indicates that the small fraction of short inserts in conventional WGS libraries may provide enough information to assess sequencer error rate. Moreover, reference mapping is required for our approach to determine the overlapping regions between forward and reverse reads, which renders it challenging to apply our method to sequencing data from a species without a reference genome available. In this work, we were not able to study the effect of sequencing conditions such as cluster density and PhiX spike-in because such parameters are not reported in publicly available datasets. Future studies on these parameters are warranted.

It should be noted that barcode-based sequencing methods such as Safe-Seq and UMI methods (designed to suppress both PCR errors and sequencer errors through redundant sequencing followed by read collapsing within read families labeled by the same barcode) are an effective experimental error suppression method, but it cannot be used to specifically measure the sequencer errors because PCR errors and sequencer errors cannot be separated by barcode-sequencing technology (Fig. 1a, b). On the other hand, because our method can provide accuracy information on the sequencer, we believe an integrative approach of our method with barcode-sequencing methods can result in a further improvement of the overall sequencing accuracy, which will be our future study focus.

In summary, we have developed a computational method that can precisely assess sequencer errors. By using a large cohort of public datasets, we discovered error patterns across platforms and among sequencers, flow cells, and tiles. We also developed software that can discover and computationally suppress such errors. We expect our method to impact the assessment, monitoring, and ultimately the improvement of sequencer accuracy.

## Methods

### Read name

The raw sequencing reads have names formatted as follows: <instrument>:<run number>:<flowcell ID>:<lane>:<tile>:<x-pos>:<y-pos> (https://help.basespace.illumina.com/articles/descriptive/fastq-files/; last accessed February 11, 2020). For example, the first record in dataset ERR3790565 has a read name of A00363:103:H3CMMDRXX:1:1101:21124:1000, which indicates that the sequencer ID is A00363, and this dataset was generated on its 103rd run, on a flow cell with ID H3CMMDRXX. This read was generated in lane 1, on tile 1101, with *x* position 21,124 and *y* position 1000. Our algorithm parses the read name to obtain information on sequencer, flow cell, and tiles according to this format.

### Public sample acquisition

We tested our method on public datasets from NCBI SRA (https://www.ncbi.nlm.nih.gov/sra). We searched NCBI SRA datasets by using the following filters: (1) species is human; (2) data is paired end; (3) platform is either HiSeq, NextSeq, or NovaSeq; (4) read length is at least 70 bps (to allow overlap between forward and reverse reads); and (5) data is deposited between January 2015 and December 2019. We discovered that many datasets do not have read names in NCBI SRA, possibly to save storage space, rendering the dataset unsuitable for our purpose (Additional file 1: Supplementary Notes 2-3). To avoid downloading datasets unsuitable for our analysis, we manually checked several samples per study by using the NCBI SRA web application (Additional file 1: Supplementary Notes 2-3). A study was excluded if it failed this manual check (Additional files 3, 4, 5: Tables S2, S3, S4, Fig. 1d). It should be noted that this procedure may result in missing datasets from studies with heterogeneous read name information.

A dataset may have a shorter read length. For example, dataset SRR10388700 has a read length of 36, so the forward-reverse overlap is minimal (Additional file 1: Supplementary Note 3). A significant fraction (19%) of datasets from the NextSeq platform were excluded by this filter (Fig. 1d).

A dataset with few reads is not informative for our analysis. For example, NovaSeq dataset SRR8717673 has only 31.6 million bases. We, therefore, excluded SRA runs when the number of bases is < 500 million for HiSeq and < 100 million for NextSeq and NovaSeq. Thirty-three percent of NextSeq datasets were excluded by this filter (Fig. 1d).

For HiSeq sequencers, there are a few studies with a large number of datasets, such as the study SRP214023 with 600 datasets. We decided to exclude such “very large” studies (those with > 50 datasets) so that we can have a broader representation of different research institutions. Studies < 10 datasets were excluded as well. Some datasets, such as study SRP215355, were found to have some samples with lost read names and therefore a study size < 10. After this filter, 2830 HiSeq datasets were included in our analysis (Fig. 1d, Additional file 3: Table S2).

### Flow cell layout information

We obtained the physical layout information for HiSeq, NextSeq, and NovaSeq as described in Additional file 1: Supplementary Note 4-6 (as of February 11, 2020). Such information was used to generate Fig. 1e–g.

### Algorithm description

To calculate the sequencer error rate (sER), we utilized mismatches in the overlapping regions between forward-reverse read pairs. We first ensure the read pairs have good sequencing quality by using the method as described previously [8]: (1) a read with poor mapping quality (MAPQ < 55 or MAPQ > 254) is discarded, (2) the read must not have complex alignments (the CIGAR string has a pattern of digits followed by the letter “M,” i.e., matches regular expression /^\d+M$/), (3) the overall Phred quality of the read must be good (< 5% of bases to have Phred quality score < 20), and (4) a base with Phred quality score < 30 is excluded even if its read is included. Because our method relies on forward-reverse read pairs, in this work, we required the reads must be properly paired. In addition, (5) the first five base pairs of both forward and reverse reads were removed for the well-known quality drop at read end [8]. To determine the mutation type (i.e., one of the 12 possible misincorporations), we (6) first performed allele counting by using the previously published CleanDeepSeq algorithm [8], and determined the genotypes of all genomic positions with depth > 10×; genomic sites with a dominant allele (allele fraction > 95%) are used to calculate errors. The algorithm is implemented in C++.

### Reference DNA library: COLO829 dilution dataset

To compare the effect of different sequencers as well as outlier-tile removal on the same reference DNA library, we took advantage of the COLO829 dilution dataset (NCBI SRA: PRJNA474341) generated previously [8]. Briefly, the melanoma cell line COLO829 (ATCC CRL-1974) and its matched normal cell line COLO829BL (ATCC CRL-1980; derived from peripheral blood of the same patient) have been well studied for somatic DNA variants and are proposed to serve as a reference standard for cancer genome sequencing [25, 26]. A dilution experiment was performed previously [8] to study the error profiles in deep next-generation sequencing datasets, where DNA from melanoma cell line COLO829 was mixed with DNA from the normal cell line COLO829BL at low concentrations of 1:1000 and 1:5000 to mimic the low allele fraction scenario. To generate spike-in controls, 19 known somatic substitutions were analyzed by amplicon sequencing (with a flanking region ~ 100 bps for each marker) at > 1,000,000 depth [8]. In this dataset, the same DNA library was sequenced at SJ (St. Jude Children’s Research Hospital, sequencer identifier A00214) and HAIB (HudsonAlpha Institute of Biotechnology, sequencer identifier A00363) independently.

Interestingly, upon downloading, we found that the read name of our submitted dataset (PRJNA474341) was also lost during submission. To enable reproducibility of our results, we have re-uploaded all relevant FastQ files to European Nucleotide Archive (ENA, https://www.ebi.ac.uk/ena) with accession number PRJEB35986 and with the read names preserved. The sample ID mappings are provided in Additional file 15: Table S14. Datasets with PCR amplification using NEB Q5 High-Fidelity DNA polymerase and sequenced with NovaSeq were analyzed in this work.

### Data source, filtering, and processing

All public data (Additional files 3, 4, 5, 15: Tables S2, S3, S4, S14) were downloaded from NCBI SRA by using the SRA Toolkit (v2.8.1.3; https://www.ncbi.nlm.nih.gov/books/NBK158900/). The downloaded FastQ files were mapped to hg19 as previously described [8] by using bwa (0.7.12-r1039) with the option “aln.” A total of 1663 whole-genome sequencing data (Additional file 3: Table S2, rows 22,029–23,691) are downloaded from a previous St. Jude LIFE (SJLIFE) study [27] which is accessible at St. Jude Cloud (https://platform.stjude.cloud/requests/cohorts). For example, the outlier sequencer E00332 in Fig. 2b was used for this cohort. All other relevant data are included in the article or supplementary files.

Because sequencing errors are rare, a large number of overlapped base pairs between forward and reverse reads are needed to obtain reliable estimates of error rates. For this purpose, we required a flow cell to have at least 2,000,000 overlapping base pairs to be included in the analysis (e.g., Additional file 1: Fig. S2). The same threshold was used at the sample level for Additional file 1: Fig. S1. This threshold is reduced to 1,000,000 when analyzing the surface-level error rates in Fig. 2c, d, Additional file 1: Fig. S3, S4, S5.

Within each platform, such as HiSeq, there could be differences among sub-models, such as HiSeq 2000/4000. For example, the flow cells could have a different number of tiles per swath (Additional file 1: Supplementary Note 4-6). To account for this, we generated Additional file 1: Fig. S4 and S5 by using flow cells with the most frequent number of tiles (HiSeq, 24; NextSeq, 12; NovaSeq, 78).

### Data analysis with error correction methods

Files in FastQ format were corrected by using Lighter and Musket methods with the reported optimum k-mer sizes, i.e., 30 and 28, respectively [22]. Both corrected and uncorrected FastQ files were aligned to hg19 using BWA aln [28]. The pileup summary of aligned reads was calculated using LoFreq *plpsummary* command with parameters -Q 30 -q 30 -m 55 -d100000000 [29].

### SequencErr on St. Jude Cloud

An end-to-end pipeline deployable through a graphical point-and-click interface is available on St. Jude Cloud (https://platform.stjude.cloud/workflows/sequencerr). Academic users can create an account in St. Jude Cloud and run this pipeline without restrictions.

## Supplementary Information

**Additional file 1: Supplementary Figure S1.** Minimal sample effect for calculating error rates for flow cells. **Supplementary Figure S2.** Flow cell-level error rate of all sequencers analyzed in this study. **Supplementary Figure S3.** Physical location pattern of tile-level error rates across flow cells in an outlier sequencer. **Supplementary Figure S4.** Prevalence of outlier tiles at flow cell level. **Supplementary Figure S5.** Positional pattern of problematic tiles. **Supplementary Figure S6.** Comparison of sequencers by using a common reference DNA library. **Supplementary Figure S7.** Effect of removing outlier tiles on the overall sequencing error rate. **Supplementary Figure S8.** Effect of removing outlier tiles evaluated by fold change. **Supplementary Figure S9.** Benchmarking SequencErr with FastQC. **Supplementary Figure S10.** Effect of DNA sequencing features on sequencer error rate. **Supplementary Figure S11.** Comparison of SequencErr with error correction methods. **Supplementary Figure S12.** Application of SequencErr on non-human dataset (SARS-CoV-2). **Supplementary Note 1.** Illustration of flowcell architecture. **Supplementary Note 2.** Manually checking read name information from NCBI SRA. **Supplementary Note 3.** Manually checking NCBI SRA database for the suitability of public datasets for our analysis. **Supplementary Note 4.** Flow cell layout of HiSeq. **Supplementary Note 5.** Flow cell layout of NextSeq. **Supplementary Note 6.** Flow cell layout of NovaSeq.

**Additional file 2: Supplementary Table S1.** List of NCBI SRA (https://www.ncbi.nlm.nih.gov/sra) studies and associated platform, research institute and country.

**Additional file 3: Supplementary Table S2.** HiSeq datasets. Publicly accessible studies deposited in NCBI SRA (https://www.ncbi.nlm.nih.gov/sra) were reviewed to account for lost read names.

**Additional file 4: Supplementary Table S3.** NextSeq datasets. Publicly accessible studies deposited in NCBI SRA (https://www.ncbi.nlm.nih.gov/sra) were reviewed to account for lost read names.

**Additional file 5: Supplementary Table S4.** NovaSeq datasets. Publicly accessible studies deposited in NCBI SRA (https://www.ncbi.nlm.nih.gov/sra) were reviewed to account for lost read names.

**Additional file 6: Supplementary Table S5.** Sample effect is minimal on flow cell-level analysis.

**Additional file 7: Supplementary Table S6.** Flow cell-level sequencer error rate.

**Additional file 8: Supplementary Table S7.** All flow cells affected in an outlier sequencer.

**Additional file 9: Supplementary Table S8.** Surface-level sequencer error rate.

**Additional file 10: Supplementary Table S9.** Extent of outlier tiles.

**Additional file 11: Supplementary Table S10.** Prevalence of outlier tiles at flow cell level.

**Additional file 12: Supplementary Table S11.** Outlier tiles.

**Additional file 13: Supplementary Table S12.** Tile-level sequencer error rate estimated using a common reference DNA library.

**Additional file 14: Supplementary Table S13.** Sequencer effect and outlier tile effect on overall error rate of a common reference DNA library.

**Additional file 15: Supplementary Table S14.** Sample names of a dataset generated in different sequencing centers by using a common reference DNA library.

**Additional file 16:** Review history.

## Data Availability

All data used in this work are from published resources. See the complete list of accession numbers in Additional files 2, 3, 4, 5, 15: Tables S1, S2, S3, S4, S14. The source code of the SequencErr [30] and all programs used to generate figures [31] have been deposited in Zenodo. The SequencErr program has been made publicly available in the SJ Cloud (https://platform.stjude.cloud/workflows/sequencerr) for non-profit research uses.
