# Supplementary material for: SequencErr: measuring and suppressing sequencer errors in next-generation sequencing data
Source: Genome Biol. 2021 Jan 25;22:37. doi: 10.1186/s13059-020-02254-2 (PMC7829059; doi:10.1186/s13059-020-02254-2)
Supplement: Supplementary file 1 — Additional file 1: Supplementary Figure S1. Minimal sample effect for calculating error rates for flow cells. Supplementary Figure S2. Flow cell-level error rate of all sequencers analyzed in this study. Supplementary Figure S3. Physical location pattern of tile-level error rates across flow cells in an outlier sequencer. Supplementary Figure S4. Prevalence of outlier tiles at flow cell level. Supplementary Figure S5. Positional pattern of problematic tiles. Supplementary Figure S6. Comparison of sequencers by using a common reference DNA library. Supplementary Figure S7. Effect of removing outlier tiles on the overall sequencing error rate. Supplementary Figure S8. Effect of removing outlier tiles evaluated by fold change. Supplementary Figure S9. Benchmarking SequencErr with FastQC. Supplementary Figure S10. Effect of DNA sequencing features on sequencer error rate. Supplementary Figure S11. Comparison of SequencErr with error correction methods. Supplementary Figure S12. Application of SequencErr on non-human dataset (SARS-CoV-2). Supplementary Note 1. Illustration of flowcell architecture. Supplementary Note 2. Manually checking read name information from NCBI SRA. Supplementary Note 3. Manually checking NCBI SRA database for the suitability of public datasets for our analysis. Supplementary Note 4. Flow cell layout of HiSeq. Supplementary Note 5. Flow cell layout of NextSeq. Supplementary Note 6. Flow cell layout of NovaSeq. [file 13059_2020_2254_MOESM1_ESM.docx]

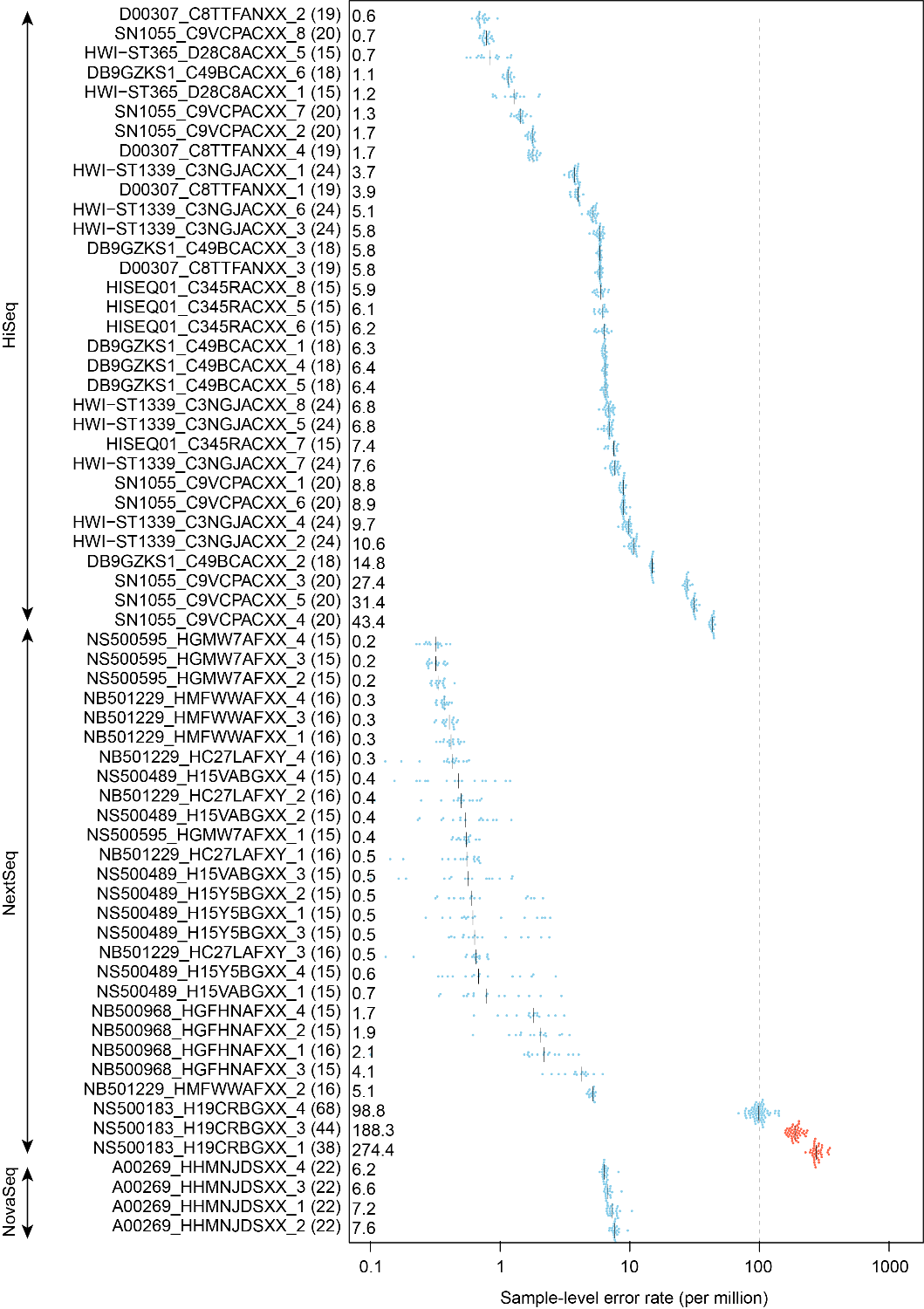


**Fig S1. Minimal sample effect for calculating error rates for flow cells.** Shown are sample-level error rates (x-axis; per million; Method) for samples pooled in the same sequencing lane of the same flow cell (y-axis). Because a typical flow cell has multiple lanes, and a common practice is to pool samples at lane level, here we grouped samples at lane level, instead of flow cell level. The row names are formatted as “Sequencer_Flowcell_Lane (number of samples)”. For example, the first row is identified as “D00307_C8TTFANXX_2 (19)”, which means the data is from sequencer D00307, with flow cell C8TTFANXX, at lane 2, where 19 samples were pooled together. Medians are indicated by a vertical line and in the left margin of the figure. Only samples with >1 million overlapping bases per lane were included.


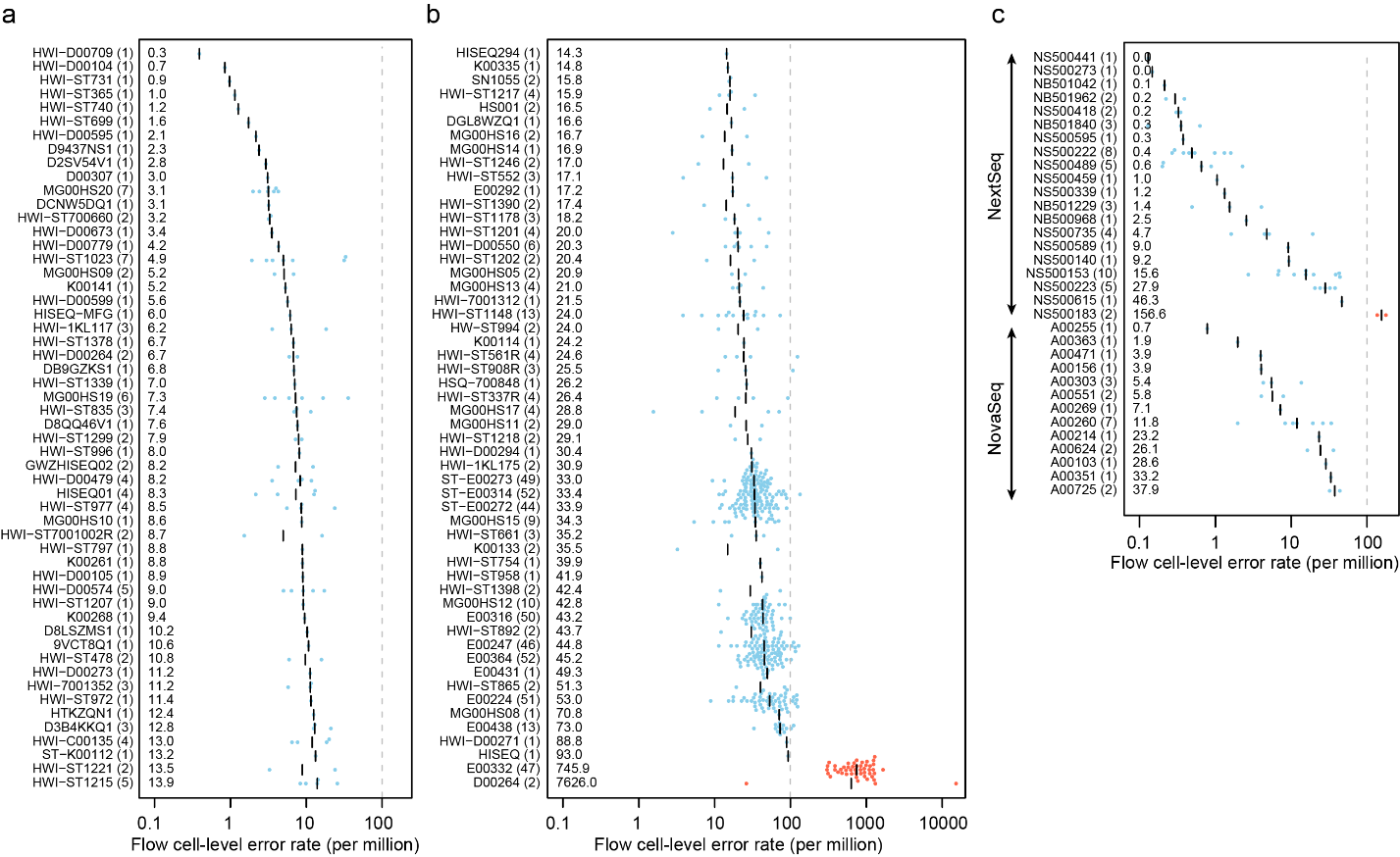


**Fig S2. Flow cell-level error rate of all sequencers analyzed in this study.** Shown are flow cell-level error rates for (a,b) HiSeq and (c) NextSeq and NovaSeq. Sequencer identifiers are indicated with number of flow cells in parenthesis. Medians across all flow cells for each sequencer are given in the left margin and indicated by vertical bars. Two HiSeq sequencers (E00332 and D00264) and one NextSeq sequencer (NS500183) with median error rate greater than 100 pm are highlighted in red.


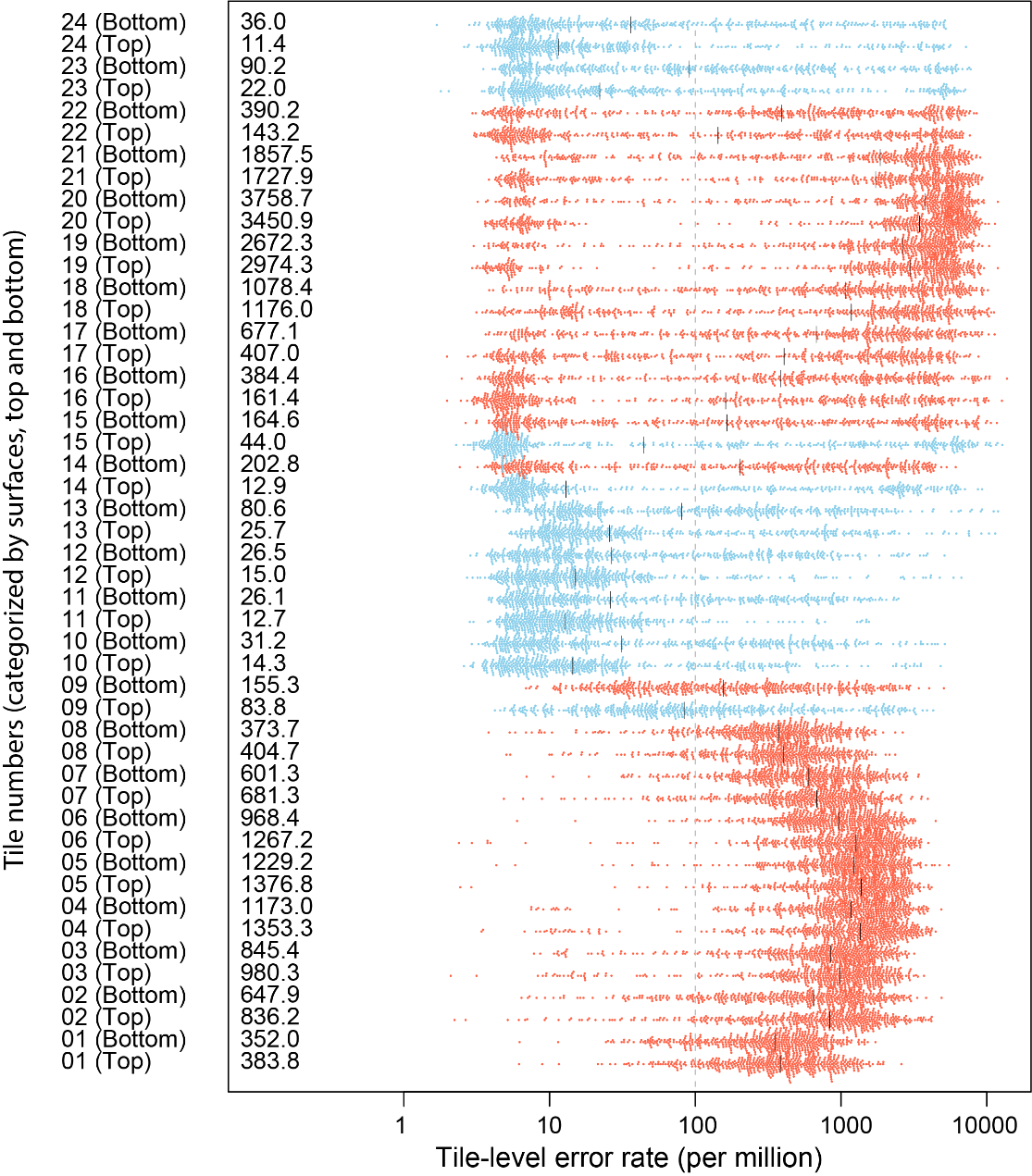


**Fig S3. Physical location pattern of tile-level error rates across flow cells in an outlier sequencer.** Shown are tile-level error rates across 47 flow cells for HiSeq sequencer E00332. Tile locations (from 01 to 24) are extracted according to Supplementary Note 2 across all flow cells. Note the quality drop in tile numbers 01-08 and 16-22.


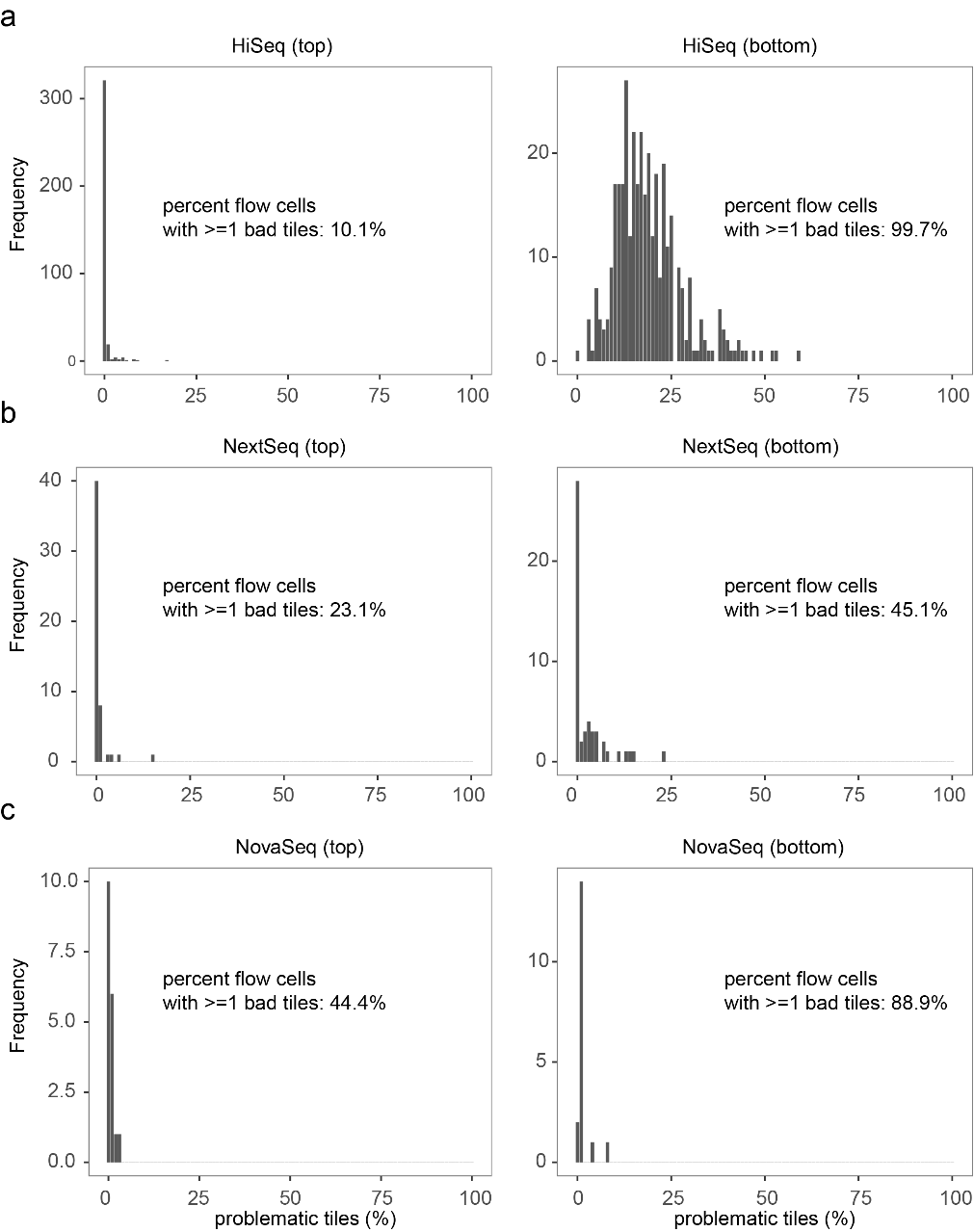


**Fig S4. Prevalence of outlier tiles at flow cell level.** Show is the frequency of flow cells (y-axis) as a function of the percentage of problematic tiles (x-axis) for (a) HiSeq, (b) NextSeq, and (c) NovaSeq, stratified by top (left panels) and bottom (right panels) surfaces. Percentage of flow cells with 1 or more problematic tiles is indicated for each panel.


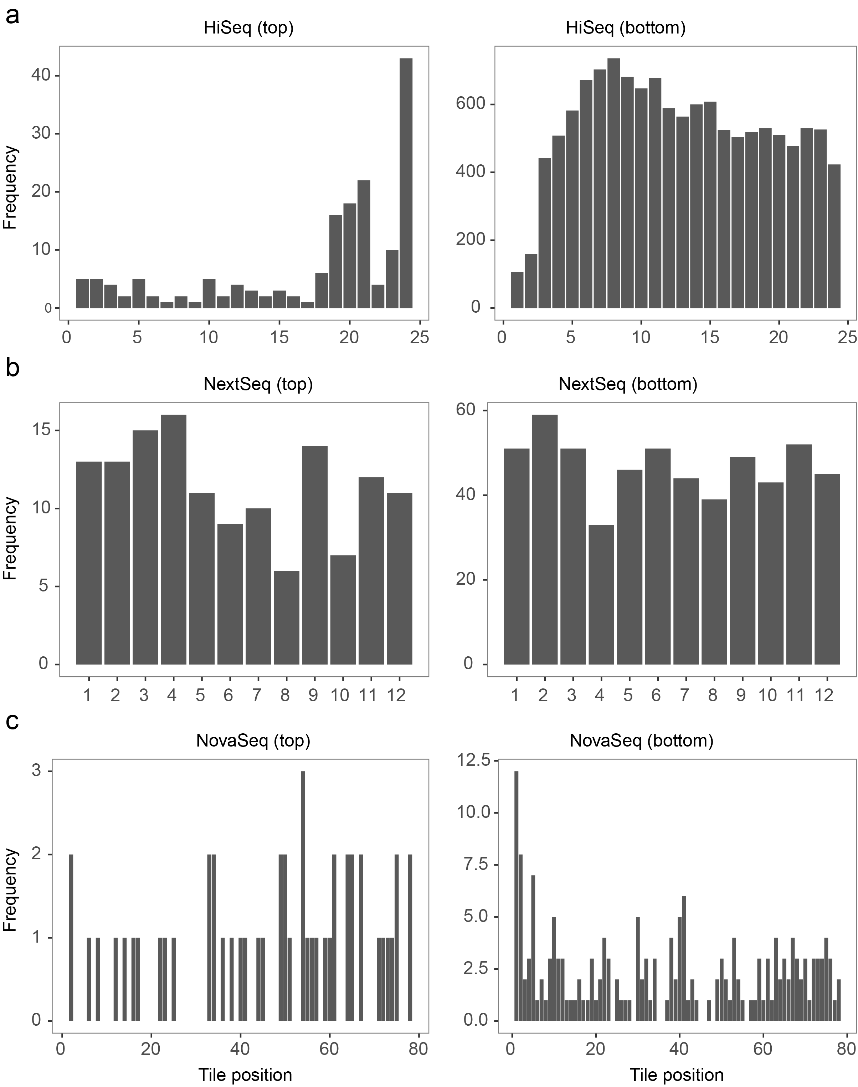


**Fig S5. Positional pattern of problematic tiles.** Shown are frequency (y-axis) of problematic tiles at positions (x-axis) defined for each flow cell type, for (a) HiSeq, (b) NextSeq and (c) NovaSeq. The data is stratified into top (left panels) and bottom (right panels) surfaces.


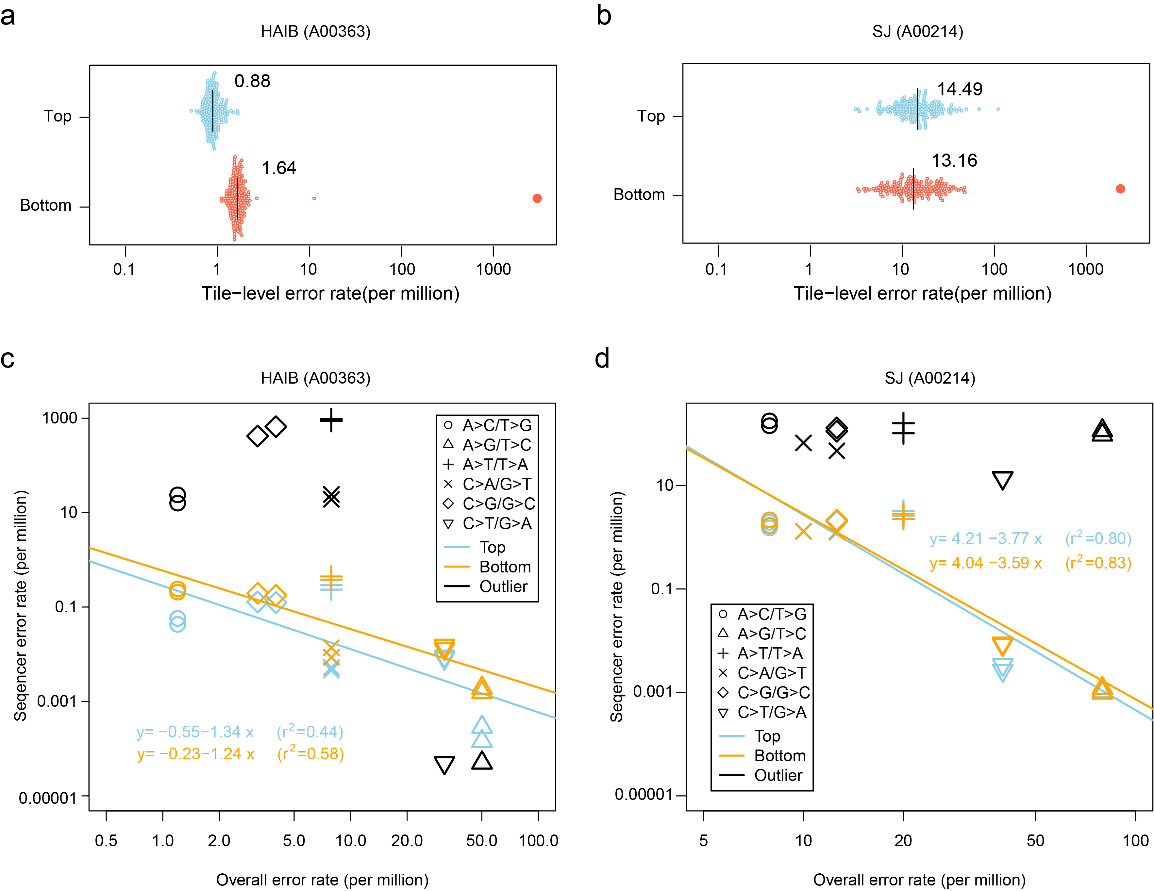


**Fig S6. Comparison of sequencers by using a common reference DNA library.** (a) The tile-level error rate in HAIB NovaSeq sequencer A00363 demonstrate a surface dependency. (b) Lack of surface dependency in SJ NovaSeq sequencer A00214. Medians are indicated by a horizontal line. Outlier tiles (>100 pm) are indicated with a large solid dot. The error rate is calculated by using a common DNA library of the COLO829 dilution experiment (Method) for sequencing. (c-d) Comparison of error rates of 12 misincorporation types between sequencer (y-axis) and overall sequencing outcome (x-axis; Eq. 4), which is a product of sequencer and PCR error rates in the dataset generated by (c) HAIB sequencer A00363 and (d) SJ sequencer A00214. Here the flow cell tiles are categorized into three groups: outliers (error rate > 100 pm, panels a-b); non-outliers in the top surface (Top); and non-outliers in the bottom surface (Bottom). Regression lines and corresponding adjusted R-squared values are indicated. The negative slope indicates an inverse correlation of error rates among error types. For example, the overall error rate of A>C/T>G is much lower than that of A>G/T>C, while the sequencer error rate of A>C/T>G is much higher than that of A>G/T>C, indicating that A>C/T>G errors are mostly induced by the sequencer, while A>G/T>C errors are mostly incurred during PCR amplification.


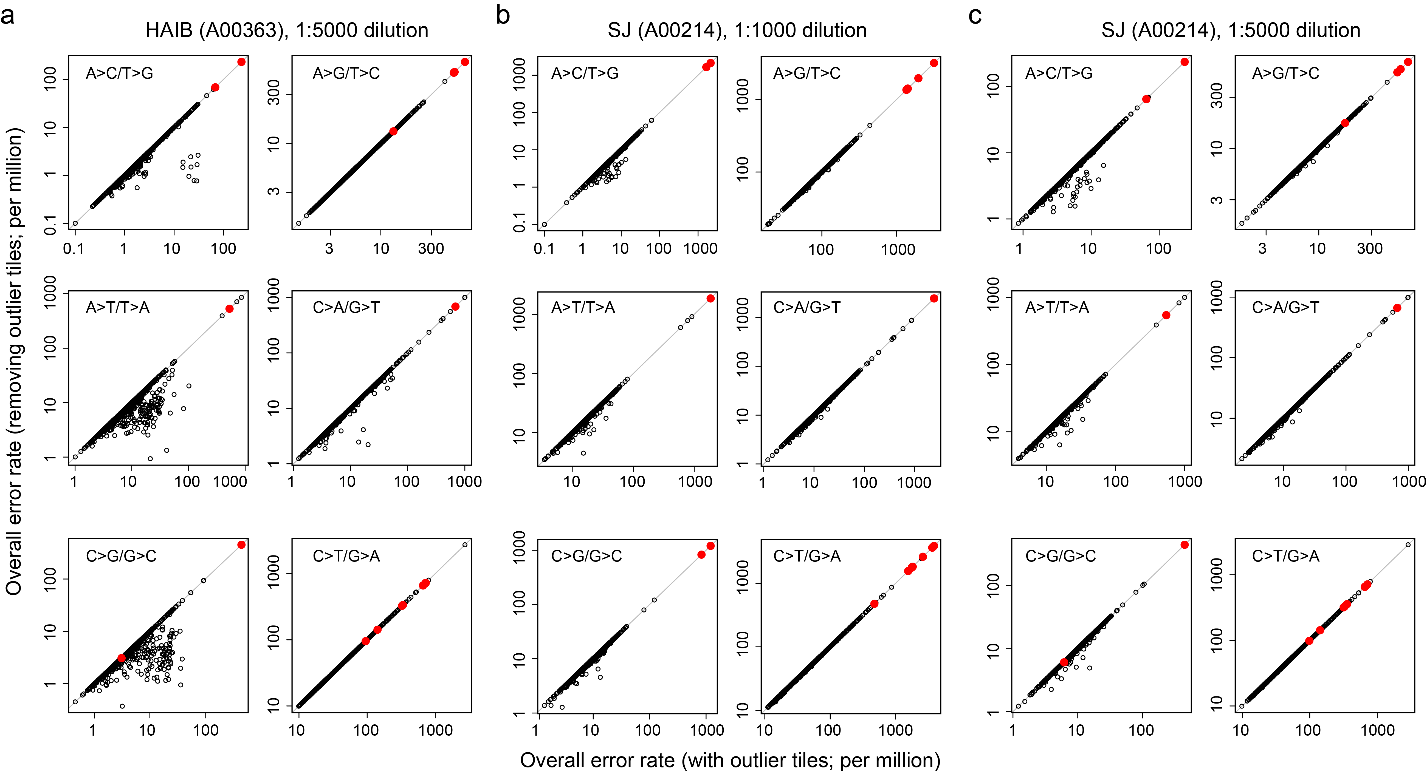


**Fig S7. Effect of removing outlier tiles on the overall sequencing error rate.** Shown are (a) the HAIB dataset, 1:5000 dilution, and the SJ dataset, (b) 1:1000 and (c) 1:5000 dilution, defined in **Fig. 3a**. Each dot represents the site-specific error rate of given misincorporation types with (x-axis) and without (y-axis) the outlier tiles. Red dots: spike-in true mutations (Method). Diagonal (no change) is indicated by gray lines. Sequencer identifiers are indicated in each panel. Error rates are given in errors per million (pm).


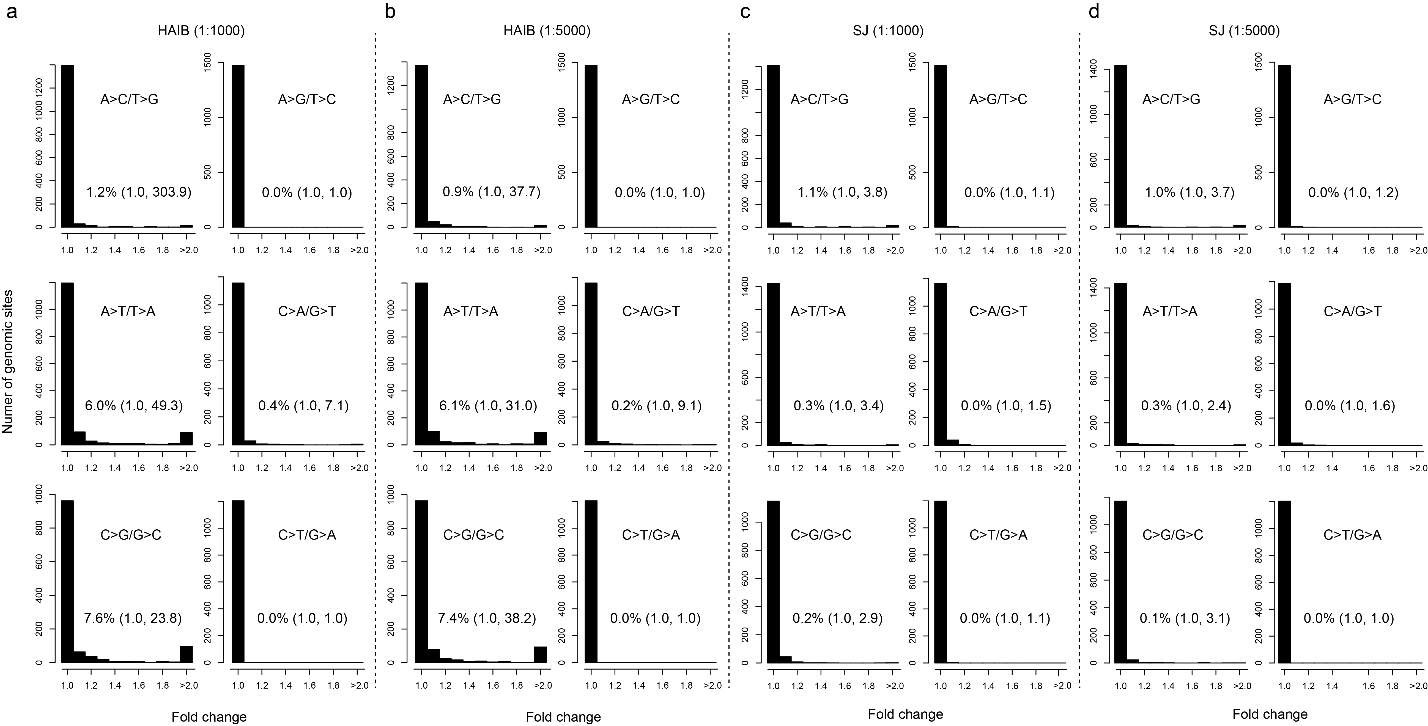


**Fig S8. Effect of removing outlier tiles evaluated by fold change.** Fold-change distribution of overall error rates at genomic site-level. The x-axis gives the fold change upon removing outlier tiles while the y-axis represents the number of genomic sites in the designed amplicon regions in the COLO829 dilution dataset (Method). The data is categorized according to misincorporation types in six panels for four dilutions: HAIB (a) 1:1000 and (b) 1:5000; and SJ (c) 1:1000 and (d) 1:5000. In each panel, the percentage of genomic sites with >2-fold error rate suppression is indicated along with range of the error rate changes. For example, 6% A>T/T>A misincorporations have >2-fold error rate reduction in the HAIB 1:1000 dataset, and the maximal error rate change is 49.3-fold.


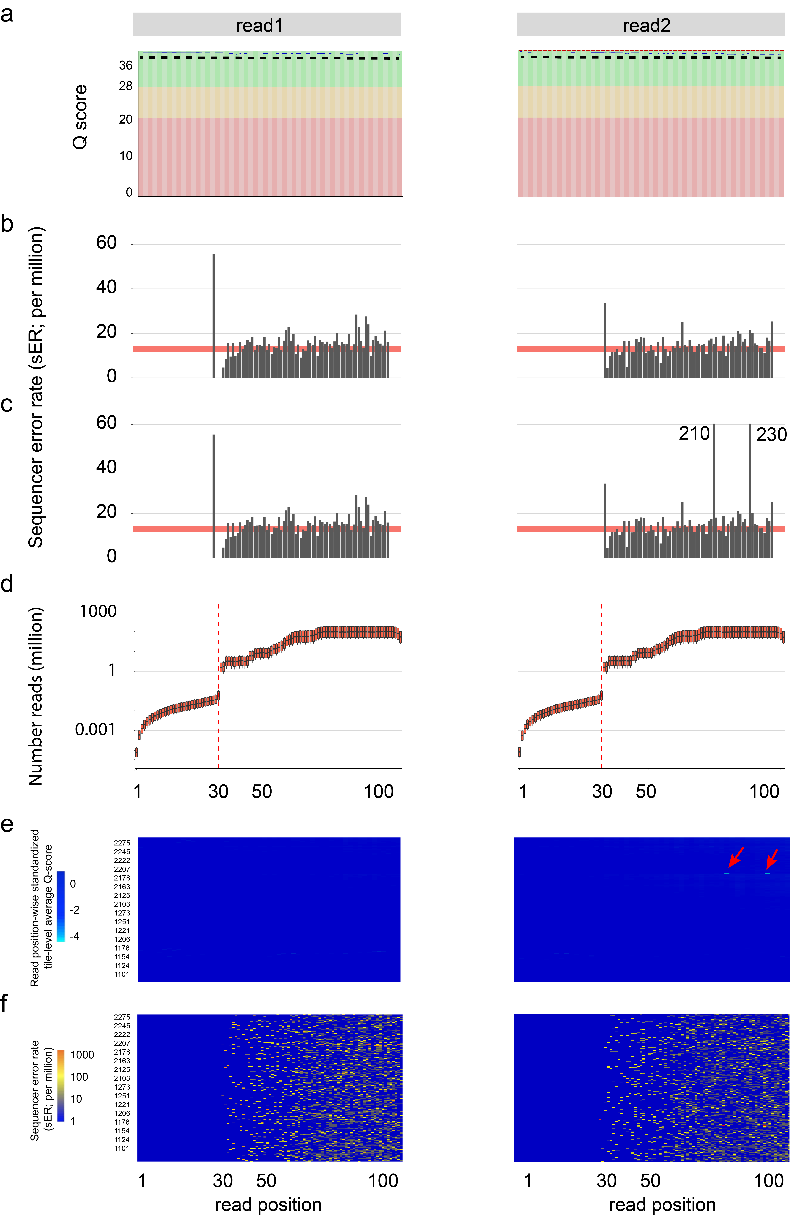


**Fig S9. Benchmarking SequencErr with FastQC.** (a). Phred score distribution of read 1 and read 2 generated by FastQC from a representative sample (NCBI SRA ERR3781298). This dataset is considered high quality by FastQC analysis. Thick blue line indicates the median Phred score at given position (all above Phred score 36), and a thick dashed black line indicates the lower bound. (b) SequencErr analysis of the same dataset. Error rate (sER) of ~11 per million (thick red line) is observed along with sequencing cycles 30-100, without obvious difference between read 1 and read 2. (c) Effect of outlier tiles. By including the outlier tile (# 2201), cycles 77 (sER=210) and 92 (sER=230) in read 2 demonstrate elevated error rate. (d) The first 30 base pairs are not well covered (<0.1 million depth) due to limited read 1 and read 2 overlap, which depends on study design of insert size and actual read length. This explains the outlier error rate profile (mostly 0, a high number in position 30) from position 1 to position 30 in panels b and c. We also show tile-level quality based on FastQC output (e) and SequencErr output (f). In FastQC figure (panel e), the average Q score data (in tile × read position format) were position-wise standardized by using formula x*_t_*_,_*_i_*-µ*_i_*, where µ*_i_* is the average Q score of all tiles at read position *i*, and x*_t_*_,_*_i_* is the average Q score at read position *i* of tile *t*. In panel e, red arrows indicate poor Q score of tile # 2201 at read positions 76-77 (score: -3.3) and 92-93 (score: -3.59), which is consistent with our SequencErr results (panel c).


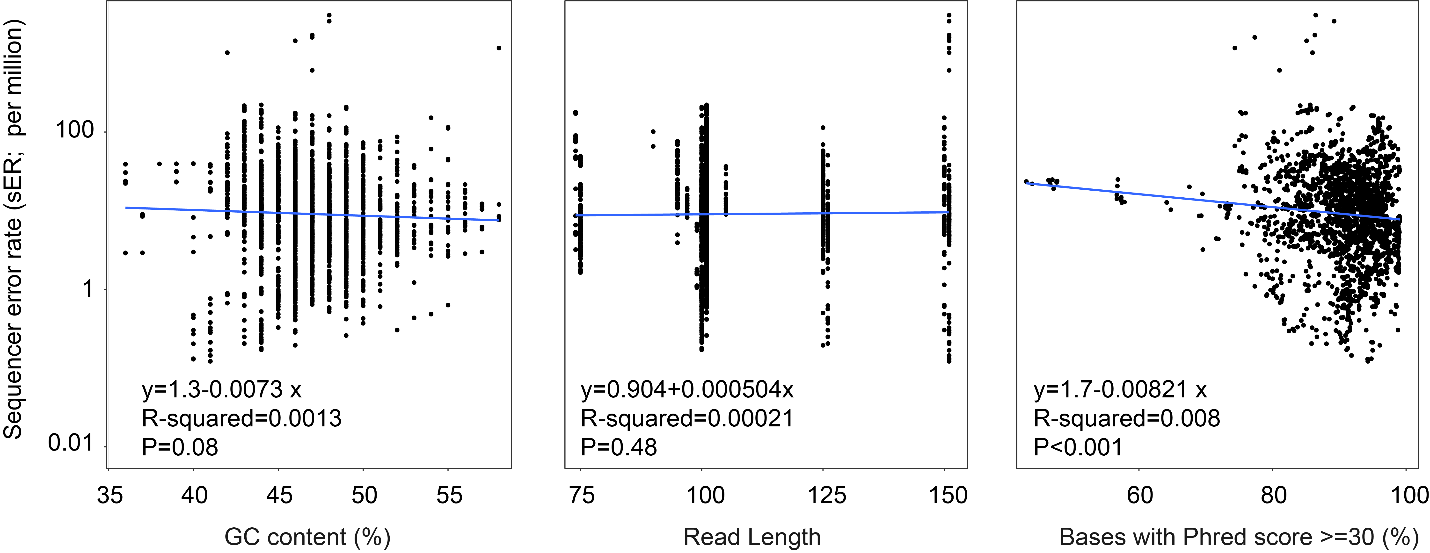


**Fig S10.** **Effect of DNA sequencing features on sequencer error rate.** Shown are the effect of GC content (a), read length (b), and overall Q30 percentage (c). A linear model was fit for each feature, with R-squared and corresponding P value indicated.


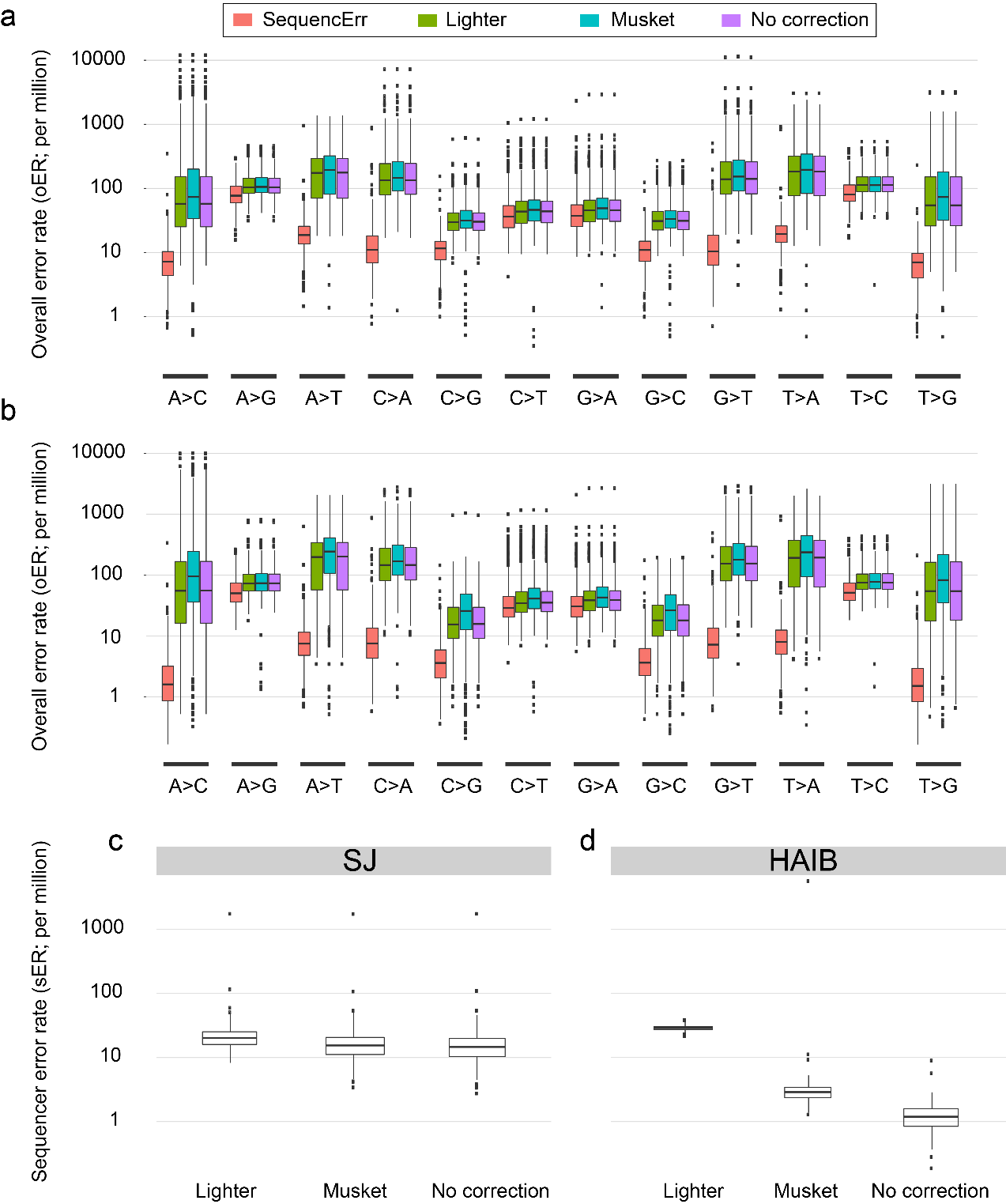


**Fig S11.** **Comparison of SequencErr with error correction methods.** Shown are overall error rate (oER) distribution of SequencErr (red), Lighter (green), Musket (blue), and raw data without error correction/suppression (No correction, purple), stratified by 12 possible nucleotide changes, for dataset generated by St. Jude (a) and HudsonAlpha (b). Also shown are sequencer error rate (sER) estimated by overlapping read pair method for data generated by St. Jude (c) and HudsonAlpha (d). In (c) and (d), sequencer error rate becomes higher (~10-fold) than no correction (SequencErr measurement), indicating over-correction.


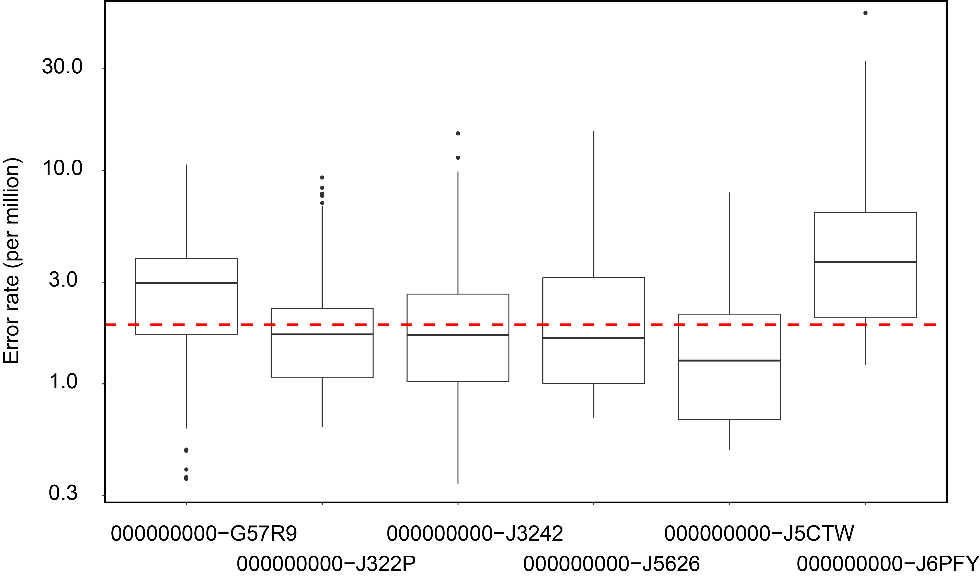


**Fig S12. Application of SequencErr on non-human dataset (SARS-CoV-2)**. Tile-level boxplot of sequencer error rate (y-axis) from 6 flowcells (x-axis) appears to be comparable to that (red dashed line) of human genome sequencing.

Supplementary Note 1. Illustration of flowcell architecture.

Illustrated are top/bottom surface, lane, swath, and tiles, and how such information are encoded in read names.


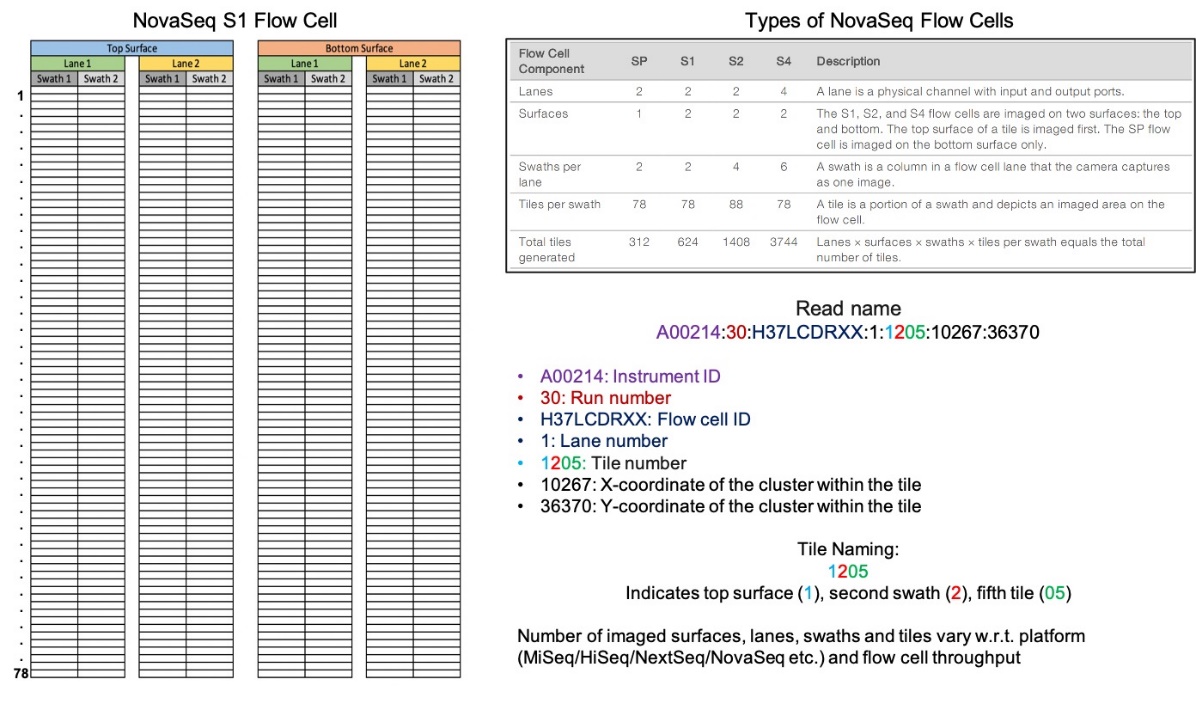


Supplementary Note 2. Manually checking read name information from NCBI SRA

Taking NextSeq datasets as an example, we started with 33,160 records (Additional File 4) and reviewed the data for inclusion in this study. Because we need large datasets to evaluate the low sequencer error rates, we excluded 11,047 datasets with size <100MB (average 10 MB). We next filtered 6,175 datasets with short reads (<70 bp for either forward or reverse reads) since our method relies on overlapping forward and reverse reads. Because our method also relies on the availability of read names that provide information regarding instrument, flow cell, surface, lane, swath and tile, we manually checked a few datasets for such information. Surprisingly, many datasets have no read names. To save the effort in downloading data that cannot be used for our purpose, we manually reviewed each dataset (Supplementary Note 3) at https://www.ncbi.nlm.nih.gov/sra. Clearly, it is impractical to review every entry of thousands of datasets by this approach. We therefore chose to review only one dataset per study, with the assumption that the data submission procedure is relatively homogeneous. As it turned out, only 2.6% of publicly accessible NextSeq datasets can be used for our study purpose. A similar strategy was used to select NovaSeq and HiSeq datasets.


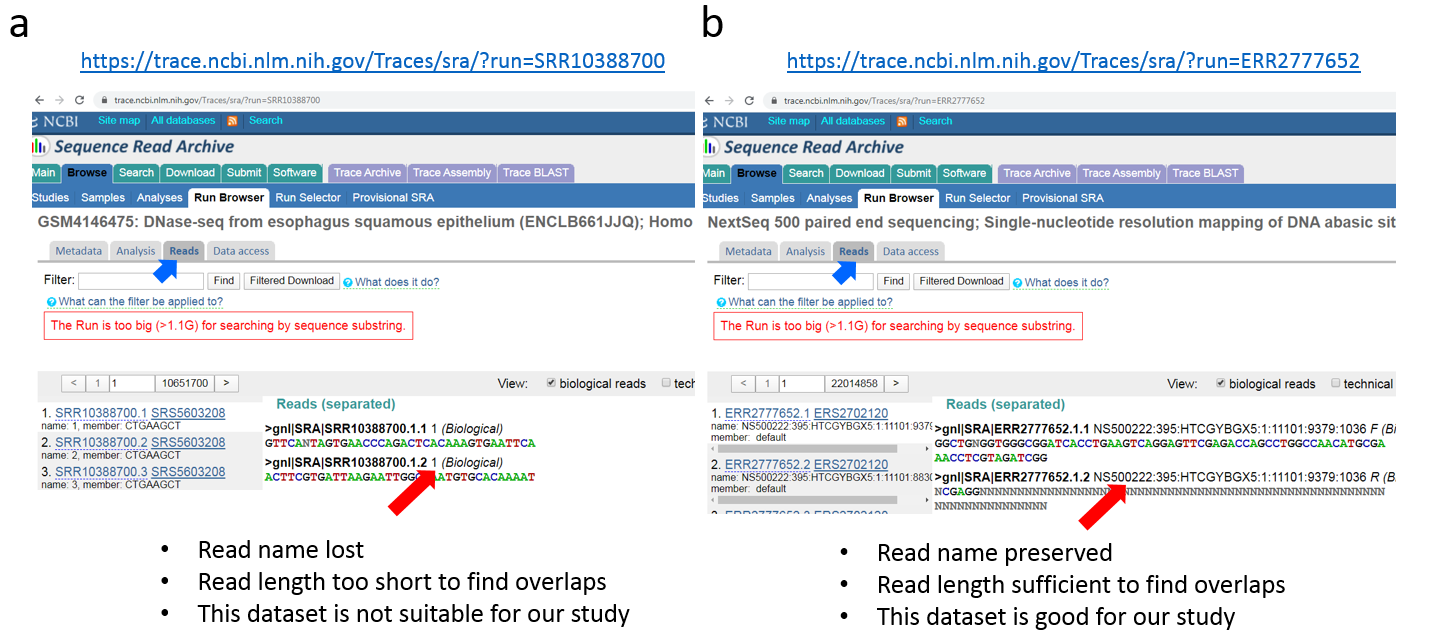


**Supplementary Note 3. Manually checking NCBI SRA database for the suitability of public datasets for our analysis.** (**a**) Dataset SRR10388700 is unsuitable for our analysis because 1) the read name is missing; 2) the read length of 36 bps is too short to find overlap between forward and reverse reads. (**b**) Dataset ERR2777652 is good for our study, because the read name is preserved, and the read length of 75 (forward) + 75 (reverse) is sufficient to find overlaps. Accessed February 11, 2020.

**Supplementary Note 4. Flow cell layout of HiSeq**. Accessed February 11, 2020 (version: 15035786 v02) from https://support.illumina.com/downloads/hiseq_2500_user_guide_15035786.html

**Supplementary Note 5. Flow cell layout of NextSeq.** Accessed February 11, 2020 (version: (15046563 v06) from https://support.illumina.com/downloads/nextseq-500-user-guide-15046563.html

**Supplementary Note 6.** Flow cell layout of NovaSeq. Accessed February 11, 2020 (version: 1000000019358 v11) from https://support.illumina.com/downloads/novaseq-6000-system-guide-1000000019358.html

1 Ma, X. *et al.* Analysis of error profiles in deep next-generation sequencing data. *Genome Biol* **20**, 50, doi:10.1186/s13059-019-1659-6 (2019).

2 Pleasance, E. D. *et al.* A comprehensive catalogue of somatic mutations from a human cancer genome. *Nature* **463**, 191-196, doi:10.1038/nature08658 (2010).

3 Craig, D. W. *et al.* A somatic reference standard for cancer genome sequencing. *Sci Rep* **6**, 24607, doi:10.1038/srep24607 (2016).
