# Supplementary material for: SequencErr: measuring and suppressing sequencer errors in next-generation sequencing data
Source: Genome Biol. 2021 Jan 25;22:37. doi: 10.1186/s13059-020-02254-2 (PMC7829059; doi:10.1186/s13059-020-02254-2)
Supplement: Supplementary file 16 — Additional file 16: Review history. [file 13059_2020_2254_MOESM16_ESM.docx]

Review History

**First round of review**

**Reviewer 1**

**Are you able to assess all statistics in the manuscript, including the appropriateness of statistical tests used?**

Yes, and I have assessed the statistics in my report.

**Comments to author:**

"SequencErr: Measuring and suppressing sequencer errors in next generation sequencing" by Davis et al. evaluates the use of overlapping paired end reads to assess the error profiles of different Illumina sequencers, to identify sequencing runs or portions of a flow cell which have elevated error rates, and proposes an approach to lower sequencer error rates by removing data from error-prone tiles.

The accuracy of base calling is important for the detection of sequence variants, in particular for rare somatic variants where the allele frequency may be at or below the level of the sequencing error rate. A number of approaches have been developed to boost the accuracy of NGS data, including the use of information in overlapping sequencing reads (such as paired end reads in Illumina sequencing, 2d and 1d2 reads in Nanopore sequencing, or CCS reads in PacBio sequencing) and the addition of molecular tags (Unique Molecular Identifiers) to enable error correction within tagged consensus families.

It has previously been demonstrated that use of overlapping reads can reduce error rates in NGS data. See, for instance (to name just a subset of papers which have done this): https://bmcgenomics.biomedcentral.com/articles/10.1186/s12864-015-2194-9).

https://elifesciences.org/articles/03300

https://www.nature.com/articles/nmeth.1492

https://bmcbioinformatics.biomedcentral.com/articles/10.1186/s12859-017-1469-3

https://bmcgenomics.biomedcentral.com/articles/10.1186/s12864-015-1263-4

Thus, the approach of using information in overlapping paired end reads to assess and correct or eliminate sequencing errors on a per-read basis is well established in the field. However, this paper is novel (to my knowledge) in its application of this method of estimating error rates to QC sequencing runs at the level of flow cells, lanes, or tiles, and contains some interesting observations, such as the identification of outlier instruments (and even localized the elevated error rates to sections of the flow cell which recur from run-to-run). It should be noted though, that a similar view of error rates based on sequencing of PhiX and broken down by lane, tile, and surface is generated in Illumina's Sequencing Analysis Viewer (SAV) software; it would be informative to see how the error rates calculated by SequenceErr compare to the PhiX-derived estimates. Other tools such as FastQC also provide similar views of sequence quality across tiles of a flow cell. I would expect these measures to be similar and to have similar utility in identifying outlier tiles or underperforming instruments.

In addition, the following issues would need to be addressed prior to further consideration for publication:

1) There seems to be a substantial general underestimation of sequencer error rates using this method. Error rates of 10 per million would correspond to a Q-score of 50 and the results reported for the NextSeq (~1 per million) would correspond to a Q-score of ~60. These are much lower error rates (and higher inferred Q-scores) than what is typically seen with Illumina sequencing (where error rates are typically more on the order of 1000 per million; Q-score of ~30) and rival those reported using elaborate UMI-based low error sequencing approaches. This is likely due to the quality filtering that the authors are applying upstream of the read 1 vs. read 2 concordance comparison (requiring a Phred score of at least 30, etc.). This should at least be made clear in the text and figures that these are not measures of raw error rates.

2) Does this quality filtering in alignment and identification of mismatches affect the conclusions in other ways? For instance, sequence quality and error rates increase as sequencing cycles progress. The authors do not address this in this paper, but it would be useful to look at error rates within defined ranges along the length of the sequencing read (for instance 1-50 bp, 51-100 bp, 101-150 bp). This is a dimension along which one might also see differences between instruments/tiles. Due to the drop in Q-scores at later sequencing cycles, it is likely that many bases from later cycles would fail to pass the Q30 filter, also leading to an underestimate of error rates and an exclusion of this data from the analysis.

3) In addition, there are many technical factors in addition to just instrument that can affect sequence quality. While some of these, such as cluster density, PhiX concentration, and library type are likely not able to be assessed using data pulled from SRA, other factors such as read length, sequence diversity, GC content, and reported Q-score are readily accessible in the sequencing data and understanding the correlation between say, sequencer reported Q-score and error rate as assessed by comparison of overlapping paired end reads could help to add to the novelty of this work.

4) In assessing the section in which the same library sequenced across two different instruments at HudsonAlpha and St. Jude's, the authors ascribe the 10-fold difference in error rate between the two runs to differences between the instruments. However, since other factors such as cluster density and amount of PhiX can also influence the error rate. It would be useful to know how cluster density compares between the two runs. Since this data was generated by the authors, it also provides an opportunity to examine how the tile, surface, and lane error rates calculated on the sequencer using the PhiX spike-in compare to the metrics calculated using the SequencErr approach.

5) It should be noted that removing tiles may have a positive effect for some applications which rely on the accuracy of individual sequencing reads (such as the detection of rare somatic variants), but for other applications (such as standard variant calling through pileup consensus) there may well be a net loss in performance due to excluding the data from a tile. Ultimately, applying this correction on a per-read basis rather than a per-tile basis (as many of the papers listed above describe) will likely produce the best balance between accuracy and data yield.

In summary, it is not clear to me that this work represents a substantially novel approach to estimating error rates and correcting sequencing errors, but this approach does provide a means to detect error prone tiles in the absence of instrument-reported error rate data (which regrettably is typically not associated with SRA submissions). There are also some technical issues such as substantial underestimation of sequencer error rates that need to be addressed. Finally, consideration of additional factors which can affect sequence quality (such as position in the sequencing read and where possible, cluster density) would strengthen this work.

**Reviewer 2**

**Are you able to assess all statistics in the manuscript, including the appropriateness of statistical tests used?**

There are no statistics in the manuscript.

**Comments to author:**

Summary

The authors propose a computational method to measure the error rate of sequencers. The proposed method consists of computing the fraction of mismatches of base pairs from paired-end sequences. The authors perform an extensive analysis of public datasets comparing the sequencer error rate across platforms, flow cell surfaces and tiles levels, and sequencers. Their results indicate that bottom surfaces of flow cells tend to have higher sequencer error rates.

Major Comments

-The paper is overall well written. The research problem is clearly stated in the Introduction, and the proposed method and experimental evaluation are clearly described.

-Terminology needs to be clarified. For example, the common term is sequencing errors. Are sequencer errors different from sequencing errors? If so, this needs to be explained

-In the abstract "1.4% of sequencers" needs to be explained. The same sequencers probably could produce multiple samples among 3777 samples. Maybe authors mean 1.4% of 3777 samples?

-Authors mention the naive approach where the error rate is estimated based on the difference with the reference. It would be interesting to see how the results presented in the paper based on 3777 samples compares to the naive approach. Given that the results compare substantially, this can be used as a sanctification for the proposed approach. Otherwise one can argue that the naive approach is just good enough

-An additional approach able to accurately measure the error-rates is Safe-SeqS. High-fidelity sequencing protocol, known as Safe-SeqS (https://doi.org/10.1093/bioinformatics/btu295) is able to correct errors in the NGS data by grouping the reads originated from the same barcode. --Availability of original (with errors) data and error-free data will allow to accurate estimate the error rates

-Terminology needs to be clarified . Difference between platforms and sequencer needs to be clear defined

Why samples with modified read names dropped?

-Was all 3777 samples WGS samples? It would be interesting to compare the error rates between technologies (WES, WGS, RNA-Seq) potentially by sing the samples derived from the same individuals

There is a big discussion in the community on the value of the PHREAD scores? Were PHREAD scores used by the proposed method? Also what was the distribution of the PHREAD scores of bases determined as erroneous? Were there any erroneous bases which have very high PHREAD scores?

-It would be beneficial to add discussion how proposed approach compares to the existing computational error correction methods (https://genomebiology.biomedcentral.com/articles/10.1186/s13059-020-01988-3)

-Was any effect of read length observed. For example for similar technology was it more errors in sort reads compared to long?

-Did the authors observed a decrease in error rate over time for a given sequencing machine type.

-Did the author consider non human organisms? Is it expected to have a different error profile across various organisms?

-In section "Public samples acquisition" It is unclear what the authors mean by "For reproducibility". The authors use the public data not for reproducibility. But because this data is available

-Are samples names of 3777 samples are available?

-To allow a non-expert audience to easily understand the concepts of lanes, tiles, and surfaces, I would recommend including a figure to illustrate flow cells.

**Authors Response**

**Point-by-point responses to the reviewers’ comments:**

Reviewer #1: "SequencErr: Measuring and suppressing sequencer errors in next generation sequencing" by Davis et al. evaluates the use of overlapping paired end reads to assess the error profiles of different Illumina sequencers, to identify sequencing runs or portions of a flow cell which have elevated error rates, and proposes an approach to lower sequencer error rates by removing data from error-prone tiles.

The accuracy of base calling is important for the detection of sequence variants, in particular for rare somatic variants where the allele frequency may be at or below the level of the sequencing error rate. A number of approaches have been developed to boost the accuracy of NGS data, including the use of information in overlapping sequencing reads (such as paired end reads in Illumina sequencing, 2d and 1d2 reads in Nanopore sequencing, or CCS reads in PacBio sequencing) and the addition of molecular tags (Unique Molecular Identifiers) to enable error correction within tagged consensus families.

*[Response]: We agree with the referee’s comment on the recent progress in this field, especially molecular barcode method, which we consider as “experimental” approach as opposed to our “computational” approach. Because our method aims at improving the sequencing machine’s accuracy (or, at least on how to select the best machines for deep sequencing projects that requires high accuracy), we expect that an integrated approach of our method and barcode-based experiments may lead to further improvement. However, this idea itself is a big project and is out of our scope in this work.*

*We added following to Discussion: It should be noted that barcode-based sequencing methods, such as Safe-Seq and UMI methods (designed to suppress sequencing errors (both PCR errors and sequencer errors) through redundant sequencing followed by read collapsing within read families labeled by the same barcode) are an effective experimental error suppression method, but it cannot be used to specifically measure the sequencer errors because PCR errors and sequencer errors cannot be separated by barcode sequencing technology (Fig. 1a-b). On the other hand, because our method can provide accuracy information on sequencer, we believe an integrative approach of our method with barcode sequencing methods can result in a further improvement of overall sequencing accuracy, which will be our future study focus.*

It has previously been demonstrated that use of overlapping reads can reduce error rates in NGS data. See, for instance (to name just a subset of papers which have done this): https://bmcgenomics.biomedcentral.com/articles/10.1186/s12864-015-2194-9;

https://elifesciences.org/articles/03300;

https://www.nature.com/articles/nmeth.1492;

https://bmcbioinformatics.biomedcentral.com/articles/10.1186/s12859-017-1469-3;

https://bmcgenomics.biomedcentral.com/articles/10.1186/s12864-015-1263-4; Thus, the approach of using information in overlapping paired end reads to assess and correct or eliminate sequencing errors on a per-read basis is well established in the field. However, this paper is novel (to my knowledge) in its application of this method of estimating error rates to QC sequencing runs at the level of flow cells, lanes, or tiles, and contains some interesting observations, such as the identification of outlier instruments (and even localized the elevated error rates to sections of the flow cell which recur from run-to-run). It should be noted though, that a similar view of error rates based on sequencing of PhiX and broken down by lane, tile, and surface is generated in Illumina's Sequencing Analysis Viewer (SAV) software; it would be informative to see how the error rates calculated by SequenceErr compare to the PhiX-derived estimates. Other tools such as FastQC also provide similar views of sequence quality across tiles of a flow cell. I would expect these measures to be similar and to have similar utility in identifying outlier tiles or underperforming instruments.

*[Response]: We greatly appreciate the referee’s recognition of the novelty of this work in utilizing overlapping paired end reads to QC sequencing runs. Indeed, it is known that overlapping reads can be used to reduce error rate rates in NGS data.*

*We added to Introduction for the wide use of overlapping reads: We note that overlapping reads has been extensively utilized to reduce errors in the literature 14-18, and the novelty here is to use overlapping reads to investigate accuracy of sequencer, flowcells, etc.*

*However, the focus and important novelty of our work presented in this manuscript is that this principle can be used to address the important question of sequencer fidelity. As a result, we were able to make valuable discoveries such as the identification of outlier instruments and sections of flow cells as mentioned above by this referee, and also surprisingly, we were able to identify low error rates that “rival” those reported using elaborate UMI-based approaches (point 1 below by this referee).*

*Regarding PhiX (and SAV):*

*We agree that PhiX-derived estimates might be informative to compare with SequenceErr results. However, PhiX data is generally produced post installation/maintenance/upgrade, but not in a routine basis. For example, it is impossible to obtain the PhiX data for publicly available SRA datasets. Our COLO829 dataset generated by St. Jude and HudsonAlpha did not have PhiX spike-in either (see below email communication with HudsonAlpha).*

*We agree that SAV is a useful tool in inspecting sequencing runs. However, to our limited knowledge, it is rare for downstream analysis to utilize SAV. In fact, this is partly the reason why FastQC was developed as an alternative.*

*Regarding cluster density:*

*The NovaSeq’s patterned flow cells have a set number (2961 k/mm2) of nanowells at fixed locations. The instrument also reports a quality-filtering metric, called “Cluster Density PF%”. The dataset generated by HudsonAlpha has Density PF value between 2240-2460 k/mm2, while the value of St. Jude dataset is 2360-2540 k/mm2.*

*Regarding FastQC:*

*We thank the reviewer for raising this point which significantly strengthened our manuscript. Specifically, by analyzing publicly available datasets with both FastQC and SequencErr, we reached following conclusions: 1) Because the overall base pair quality distribution is good, FastQC does not provide further resolution to investigate problems. Rather, we can only conclude that the sequencing output is of high quality. On the other hand, our SequencErr method can still detect the errors, though at a low rate of ~11 per million. 2) most strikingly, by including/excluding the identified problematic tile, we were able to locate its effect into certain sequencing cycles.*

*We have added a new section “Comparison of SequencErr with FastQC” for this finding:*

*Because SequencErr is designed to understand sequencing quality, which is conceptually similar to FastQC 19—a frequently used quality control tool for sequencing datasets—we also benchmarked our method against FastQC. However, these two methods are fundamentally different in their design principle. FastQC operates on Phred scores (commonly known as Q30 percentage, which measures percentage of bases with Phred score above 30), but not the actual sequencing readout. On the other hand, SequencErr operates on actual sequencing readouts, although certain quality filters including Phred score distribution is applied to remove poor quality data. We used the previously published dataset 8 for this comparison. As seen in Fig. S9a, the FastQC evaluation shows that the Q30 percentage is >98% across all sequencing cycles, which would be considered good quality. However, by using our formula 1 at sequencing cycle level, we observed a stable error rate of ~11 per million across the sequencing cycles (Fig. S9b). Strikingly, we were able to observe the effect of problematic tiles on the sequencing cycles (Fig. S9b,c,f), which cannot be observed by reviewing FastQC output file (Fig. S9a,e).*

*Fig S9. Benchmarking SequencErr with FastQC. (a). Phred score distribution of read 1 and read 2 generated by FastQC from a representative sample (NCBI SRA ERR3781298). This dataset is considered high quality by FastQC analysis. Thick blue line indicates the median Phred score at given position (all above Phred score 36), and a thick dashed black line indicates the lower bound. (b) SequencErr analysis of the same dataset. Error rate (sER) of ~11 per million (thick red line) is observed along with sequencing cycles 30-100, without obvious difference between read 1 and read 2. (c) Effect of outlier tiles. By including the outlier tile (# 2201), cycles 77 (sER=210) and 92 (sER=230) in read 2 demonstrate elevated error rate. (d) The first 30 base pairs are not well covered (<0.1 million depth) due to limited read 1 and read 2 overlap, which depends on study design of insert size and actual read length. This explains the outlier error rate profile (mostly 0, a high number in position 30) from position 1 to position 30 in panels b and c. We also show tile-level quality based on FastQC output (e) and SequencErr output (f).*

In addition, the following issues would need to be addressed prior to further consideration for publication:

1) There seems to be a substantial general underestimation of sequencer error rates using this method. Error rates of 10 per million would correspond to a Q-score of 50 and the results reported for the NextSeq (~1 per million) would correspond to a Q-score of ~60. These are much lower error rates (and higher inferred Q-scores) than what is typically seen with Illumina sequencing (where error rates are typically more on the order of 1000 per million; Q-score of ~30) and rival those reported using elaborate UMI-based low error sequencing approaches. This is likely due to the quality filtering that the authors are applying upstream of the read 1 vs. read 2 concordance comparison (requiring a Phred score of at least 30, etc.). This should at least be made clear in the text and figures that these are not measures of raw error rates.

*[Response]: We appreciate this referee’s excellent examination of the Q scores and the error probabilities. We also thank this referee’s recognition that our reported error rate “rival” those reported using elaborate UMI approaches.*

*Interestingly, even though this referee deems our reported error rates being significantly lower than that estimated by Q-scores, we believe this is actually an important result rather than an “underestimate”. This is because the validity of the Q-score (also known as PHRED score) is currently in question, as indicated by referee 2’s comment “There is a big discussion in the community on the value of the PHREAD scores”. This discrepancy also highlights the importance of our study by providing additional insights to the community that Q-score may not provide accurate estimate of sequencing error.*

*We have added following in Discussion to clarify this point: “In addition to sER, our reported overall error rate (oER) of 10-100 pm (Fig. 1hs), which includes other error sources such as PCR errors, is 10- to 100-fold lower than generally reported accuracy of next generation sequencing methods of 1000 pm (10-3) 2. This reflects the successful “suppression” of poor-quality subset of data by our novel strategy. On the other hand, this raised an interesting perspective on the base call quality scores, known as Phred score. Traditional Phred scores were calculated based on sequencing traces of A/C/G/T bases by summarizing it to features such as peak spacing, uncalled/called ratio and peak resolution 23. The feature of called/uncalled ratio is a particularly interesting parameter, in that it measures the contrast between the true base and the other three non-bases of the corresponding position. Because noise is intrinsic to any scientific measure 2, signal strength of the uncalled bases is generally not 0. However, with the improvement of chemistry and physics, it is highly likely that the signal strength (i.e., noise level) of the non-bases will approach 0. Therefore, a non-0 pseudo number might have been used in the proprietary base call algorithms to prevent floating point overflow problem. For example, the current NovaSeq platforms only report Phred score up to 42, which correspond to P value of ~10-4. Because our data indicated that the calls at many bases have error rate close to 10-5 (at least in NovaSeq), we believe a re-evaluation of current Phred score calculation is warranted by the instrument manufacturer.”*

*Moreover, we have previously demonstrated that our method “rival” those barcode-based approaches (https://genomebiology.biomedcentral.com/articles/10.1186/s13059-019-1659-6) with following conclusions: “By evaluating read-specific error distributions, we discover that the substitution error rate can be computationally suppressed to 10−5 to 10−4, which is 10- to 100-fold lower than generally considered achievable (10−3) in the current literature.” This was achieved through identification and filtering of low-quality reads, which is documented in section “Algorithm description” of this submission.*

2) Does this quality filtering in alignment and identification of mismatches affect the conclusions in other ways? For instance, sequence quality and error rates increase as sequencing cycles progress. The authors do not address this in this paper, but it would be useful to look at error rates within defined ranges along the length of the sequencing read (for instance 1-50 bp, 51-100 bp, 101-150 bp). This is a dimension along which one might also see differences between instruments/tiles. Due to the drop in Q-scores at later sequencing cycles, it is likely that many bases from later cycles would fail to pass the Q30 filter, also leading to an underestimate of error rates and an exclusion of this data from the analysis.

*[Response]: Thanks for this great point! We analyzed sequencing cycle and discovered that the fidelity decay is negligible. This is likely due to the stringent quality filtering, which resulted in up to ~30% data loss as we have reported previously.*

*We added this in a new section “Comparison of SequencErr with FastQC”: Because SequencErr is designed to understand sequencing quality, which is conceptually similar to FastQC 19—a frequently used quality control tool for sequencing datasets—we also benchmarked our method against FastQC. However, these two methods are fundamentally different in their design principle. FastQC operates on Phred scores (commonly known as Q30 percentage, which measures percentage of bases with Phred score above 30), but not the actual sequencing readout. On the other hand, SequencErr operates on actual sequencing readouts, although certain quality filters including Phred score distribution is applied to remove poor quality data. We used the previously published dataset 8 for this comparison. As seen in Fig. S9a, the FastQC evaluation shows that the Q30 percentage is >98% across all sequencing cycles, which would be considered good quality. However, by using our formula 1 at sequencing cycle level, we observed a stable error rate of ~11 per million across the sequencing cycles (Fig. S9b). Strikingly, we were able to observe the effect of problematic tiles on the sequencing cycles (Fig. S9b,c,f), which cannot be observed by reviewing FastQC output file (Fig. S9a,e).*

*Fig S9. Benchmarking SequencErr with FastQC. (a). Phred score distribution of read 1 and read 2 generated by FastQC from a representative sample (NCBI SRA ERR3781298). This dataset is considered high quality by FastQC analysis. Thick blue line indicates the median Phred score at given position (all above Phred score 36), and a thick dashed black line indicates the lower bound. (b) SequencErr analysis of the same dataset. Error rate (sER) of ~11 per million (thick red line) is observed along with sequencing cycles 30-100, without obvious difference between read 1 and read 2. (c) Effect of outlier tiles. By including the outlier tile (# 2201), cycles 77 (sER=210) and 92 (sER=230) in read 2 demonstrate elevated error rate. (d) The first 30 base pairs are not well covered (<0.1 million depth) due to limited read 1 and read 2 overlap, which depends on study design of insert size and actual read length. This explains the outlier error rate profile (mostly 0, a high number in position 30) from position 1 to position 30 in panels b and c. We also show tile-level quality based on FastQC output (e) and SequencErr output (f). In FastQC figure (panel e), the average Q score data (in tile × read position format) were position-wise standardized by using formula xt,i-µi, where µi is the average Q score of all tiles at read position i, and xt,i is the average Q score at read position i of tile t. In panel e, red arrows indicate poor Q score of tile # 2201 at read positions 76-77 (score: -3.3) and 92-93 (score: -3.59), which is consistent with our SequencErr results (panel c).*

3) In addition, there are many technical factors in addition to just instrument that can affect sequence quality. While some of these, such as cluster density, PhiX concentration, and library type are likely not able to be assessed using data pulled from SRA, other factors such as read length, sequence diversity, GC content, and reported Q-score are readily accessible in the sequencing data and understanding the correlation between say, sequencer reported Q-score and error rate as assessed by comparison of overlapping paired end reads could help to add to the novelty of this work.

*[Response]: We appreciate this excellent point. In this revision we analyzed the effect of 1) GC content; 2) read length; 3) overall Q-score distribution (measured as percent bases with Q30). We discovered that these features have a negligible effect on our error measurement, though certain features, such as read length, are constrained by publicly available datasets and should be re-evaluated when read length is getting longer.*

*We added this analysis in a new section “Effect of DNA sequencing features on sequencer error rate”: We next studied the effect of DNA sequence features on sequencer error rates, by focusing on 1) GC content; 2) read length; and 3) overall base quality, measured as percentage of bases with Phred score greater than 30 (Q30 Percentage). Because large number of samples are needed to draw robust conclusion, here we focused on 1,167 publicly available HiSeq datasets (Table S1b). As seen from Fig. S10, we did not detect significant correlation between sER score and GC content and read length features, though a marginally significant negative correlation with Q30 percentage is observed, indicating that excessive percentage of low quality bases can lead to unreliable sequencing output, even if after stringent quality filtering. This indicates that our SequencErr metric being highly robust in measuring sequencer reliability in a wide range of parameter settings.*

*Fig S10. Effect of DNA sequencing features on sequencer error rate. Shown are the effect of GC content (a), read length (b), and overall Q30 percentage (c). A linear model was fit for each feature, with R-squared and corresponding P value indicated.*

4) In assessing the section in which the same library sequenced across two different instruments at HudsonAlpha and St. Jude's, the authors ascribe the 10-fold difference in error rate between the two runs to differences between the instruments. However, since other factors such as cluster density and amount of PhiX can also influence the error rate. It would be useful to know how cluster density compares between the two runs. Since this data was generated by the authors, it also provides an opportunity to examine how the tile, surface, and lane error rates calculated on the sequencer using the PhiX spike-in compare to the metrics calculated using the SequencErr approach.

*[Response]: Excellent point. Unfortunately, the data generated from both providers did not include PhiX spike-in. I have included response from HudsonAlpha in below screenshot. This also indicates that PhiX-based error rate estimation may not be universally applicable approach.*

5) It should be noted that removing tiles may have a positive effect for some applications which rely on the accuracy of individual sequencing reads (such as the detection of rare somatic variants), but for other applications (such as standard variant calling through pileup consensus) there may well be a net loss in performance due to excluding the data from a tile. Ultimately, applying this correction on a per-read basis rather than a per-tile basis (as many of the papers listed above describe) will likely produce the best balance between accuracy and data yield.

*[Response]: We totally agree with the referee on this point. In fact, our previous work (https://genomebiology.biomedcentral.com/articles/10.1186/s13059-019-1659-6) and this submission are all targeted on developing approaches that can ultimately lead to detecting somatic mutations with very low allele fractions, for which high sensitivity coupled with deep sequencing is needed. This is a high priority research direction for the research community in the past few years which will continue into future, due to its critical applications in early cancer detection and non-invasive diagnostics, which involves ultra-deep sequencing that are fundamentally different from traditional whole-genome/whole-exome sequencing. In our previous work, we have reported that ~30% data were filtered out. On the other hand, in a UMI-based approach, at least 3X over sequencing are needed to allow majority voting, which is “wasting” more (>60%) data than our filtering of 30% data.*

*We now stress this point in Discussion: On the other hand, it should be noted our method is not designed for standard sequencing projects such as whole genome and whole exome sequencing. This is because the typical tens to hundreds of sequencing depth of whole genome and whole exome sequencing is not powered to detect variants at 1% to 0.1% or even lower frequencies – which is in fact the aim of ultra-deep (tens of thousands or more) sequencing projects such as liquid biopsy 4. In this ultra-deep sequencing scenario, the 30% data loss of our method, as reported previously 8, is not a significant concern as compared with the heavily redundant sequencing of barcode-sequencing method—for example, the >60,000X sequencing of a recent large-scale UMI method resulted in a 4,577X UMI-collapsed net depth—a 13-fold redundancy 4. It is possible that our method can be further refined to filter by reads/sequencing cycles as opposed to the current tile-based filtering, which is beyond the scope of this work.*

In summary, it is not clear to me that this work represents a substantially novel approach to estimating error rates and correcting sequencing errors, but this approach does provide a means to detect error prone tiles in the absence of instrument-reported error rate data (which regrettably is typically not associated with SRA submissions). There are also some technical issues such as substantial underestimation of sequencer error rates that need to be addressed. Finally, consideration of additional factors which can affect sequence quality (such as position in the sequencing read and where possible, cluster density) would strengthen this work.

*[Response]: In this study, by using overlapping reads, we were able to make interesting discoveries concerning the identification of outlier instruments and sections of flow cells as mentioned by this referee, and surprisingly low error rates that “rival” those reported using elaborate UMI-based approaches. Thanks to the reviewers’ comments, we now demonstrated Q scores (or PHRED scores) are not accurately estimating base call qualities, which is consistent with Referee 2’s perception. Our new method not only enables the assessment and monitoring of sequencer fidelity at tile, surface, lane and machine levels, but also provides novel insights on further improving the sequencer fidelity, such as the sections of flow cells with low qualities which will ultimately lead to improvement in the instrumentation.*

*By including new analyses suggested by the reviewers, such as benchmarking with FastQC, comparing with additional error correction methods, comparing with DNA sequence features such as GC content, read length, and overall base quality distribution, as well as non-human data, we achieved novel conclusions that further strengthened this manuscript: 1) our method provided significant novel insights than FastQC can provide; 2) our method outperforms top error correction methods; 3) our method is highly robust against GC content, read length, overall base quality distribution, and both human and non-human data.*

*Therefore, we believe our manuscript has provided the research community substantial knowledge on the fidelity of DNA sequencing methods. In addition, our method also provided immediate tool set for the DNA sequencing community to make informed decisions on their experiments, and to make best use of existing deep sequencing datasets.*

Reviewer #2: Summary

The authors propose a computational method to measure the error rate of sequencers. The proposed method consists of computing the fraction of mismatches of base pairs from paired-end sequences. The authors perform an extensive analysis of public datasets comparing the sequencer error rate across platforms, flow cell surfaces and tiles levels, and sequencers. Their results indicate that bottom surfaces of flow cells tend to have higher sequencer error rates.

*[Response]: We thank referee 2 for the recognition of our work.*

Major Comments

-The paper is overall well written. The research problem is clearly stated in the Introduction, and the proposed method and experimental evaluation are clearly described.

*[Response]: Thanks!*

-Terminology needs to be clarified. For example, the common term is sequencing errors. Are sequencer errors different from sequencing errors? If so, this needs to be explained -In the abstract "1.4% of sequencers" needs to be explained. The same sequencers probably could produce multiple samples among 3777 samples. Maybe authors mean 1.4% of 3777 samples?

*[Response]: Yes, sequencer error is different from sequencing errors, where the latter contains PCR errors etc. This is the main point of our study (Fig. 1a-c).*

*We added a new section “Comparison of overall error rate (oER) and sequencer error rate (sER) measures” to clarify this: As illustrated in Fig. 1a-c, the over error rate (oER, formula 4) is a measure of sequencing errors with a mixture of error sources, including PCR artefacts and sequencer artefacts. On the other hand, the sequencer error rate (sER, formula 1-3) is a direct measure of errors specific to sequencer. We therefore compared these measures by using previously published amplicon sequencing dataset (ENA project ID PRJEB35986) 8. In this dataset, genomic loci flanking the spike-in somatic mutations are known to be wildtype and are used to measure the oER (formula 4). On the other hand, the sequencer error rate (sER) was calculated with formula 2. Because exactly same DNA library (generated by PCR enzyme Kapa and Q5, respectively) was sequenced by different NovaSeq sequencers from different sequencing providers (SJ and HAIB) 8, it also provided unique opportunity to benchmark the instruments. Consistent with the expectation that sequencer error (sER) is a subset of overall sequencing error (oER), the measured oER is consistently higher than sER (Fig. 1h). Strikingly, data generated by SJ demonstrated a significantly (Two-sided Wilcox rank sum test P<0.01) higher oER than that generated by HAIB, indicating a strong contribution of sequencer errors. Indeed, the sequencer error rate (sER) of SJ is also significantly (Two-sided Wilcox rank sum test P<0.01) higher than that of HAIB. The consistent significantly (Two-sided Wilcox rank sum test P<0.01) lower overall error rate of Q5 than that of Kapa is consistent with our previous findings 8. This data supports the value of measuring sER because lower sequencer error rate can result in lower overall error rate and measuring sER might help choosing best sequencers for deep sequencing applications.*

*Fig. 1. Measuring sequencer error rates. (a-b) Reference DNA method, where large amounts of reference DNA are needed. This can be achieved by starting from (a) small amounts of DNA/cells (to minimize inter-molecule/cell genetic heterogeneity) followed a by large number of PCR cycles and sequencing. Alternatively, we can start from (b) large amounts of starting DNA/cells followed by a small number of PCR cycles (to minimize PCR errors) and sequencing. In both approaches, mutations/PCR errors (red dots) before sequencing can confound the sequencer error rate estimate (red triangles). (c) We interrogate the sequencer errors by focusing on discordant bases between forward and reverse reads of the same DNA segment within the overlapping regions. Such mismatches must have happened in the sequencer. (d) Public datasets produced by HiSeq, NextSeq, and NovaSeq as of December 2019. Dataset without proper read names, with very small sizes, or with very short reads (so that overlap is minimal) are not suitable for our analysis (Methods). HiSeq has the highest the most suitable datasets and we downloaded and analyzed ~50% of these. (e-g) Tile-level error rate across representative sequencers for (e) HiSeq, (f) NextSeq, and (g) NovaSeq. In each panel, a “good” sequencer (top) is illustrated with a “problematic” sequencer (bottom), where sequencer identifiers are indicated on the right. (h) Comparison of overall error rate (oER, red dots) and sequencer error rate (sER, blue dots) measurements on a common DNA library (generated by PCR enzymes Kapa and Q5) sequenced by two sequencing providers (St. Jude Children’s Research Hospital Computational Biology Genomics Laboratory (SJ) and HudsonAlpha Institute of Biotechnology (HAIB)), with two different NovaSeq sequencers. Tile arrangements are determined according to vendor documentation (Method). Tile level error rates are capped at 200 per million for visualization purposes. ***: Significant Wilcox rank sum test (two-sided) P value (<0.01).*

*In section “Comparison of sequencer error rates between platforms”, we clarified the 1.4% as following: “We identified two sequencers (E00332 and NS500183, 1.4% of 141; Fig. 2b, Table S2a) as outlier sequencers and corresponding datasets were omitted from further analyses.”*

-Authors mention the naive approach where the error rate is estimated based on the difference with the reference. It would be interesting to see how the results presented in the paper based on 3777 samples compares to the naive approach. Given that the results compare substantially, this can be used as a sanctification for the proposed approach. Otherwise one can argue that the naive approach is just good enough

*[Response]: Although it is tempting to define overall sequencing error rate with the 3777 public samples through comparison with reference genome, it is practically impossible. This is because the public datasets, generally WES/WGS, are designed to detect bona fide somatic mutations, and those true mutations can easily disrupt our overall error rate (oER) estimate which aims at sequencing errors.*

*Therefore, we performed our analysis on a specifically designed dilution experiment dataset (already published and publicly available), where cancer cells (whose somatic mutations were well defined) were diluted into normal cells (from the same patient, to avoid potential effect from germline SNPs). As a result, we were able to show 1) overall error rate (oER) is order of magnitude higher than sequencer error rate (sER); 2) the same DNA library demonstrated different oER when sequenced on different instrument (from different sequencing providers), indicating a clear contribution of instrument; 3) the instrument with higher oER also has higher sequencer error rate (sER), indicating it is caused by lower fidelity of the instrument. This result clearly indicated the measure of sequencer error rate is much needed, over the naïve approach. The instrument with lower sER should be preferred for deep sequencing applications over the sequencer with higher sER.*

*We added a section “Comparison of overall error rate (oER) and sequencer error rate (sER) measures” for this: As illustrated in Fig. 1a-c, the over error rate (oER, formula 4) is a measure of sequencing errors with a mixture of error sources, including PCR artefacts and sequencer artefacts. On the other hand, the sequencer error rate (sER, formula 1-3) is a direct measure of errors specific to sequencer. We therefore compared these measures by using previously published amplicon sequencing dataset (ENA project ID PRJEB35986) 8. In this dataset, genomic loci flanking the spike-in somatic mutations are known to be wildtype and are used to measure the oER (formula 4). On the other hand, the sequencer error rate (sER) was calculated with formula 2. Because exactly same DNA library (generated by PCR enzyme Kapa and Q5, respectively) was sequenced by different NovaSeq sequencers from different sequencing providers (SJ and HAIB) 8, it also provided unique opportunity to benchmark the instruments. Consistent with the expectation that sequencer error (sER) is a subset of overall sequencing error (oER), the measured oER is consistently higher than sER (Fig. 1h). Strikingly, data generated by SJ demonstrated a significantly (Two-sided Wilcox rank sum test P<0.01) higher oER than that generated by HAIB, indicating a strong contribution of sequencer errors. Indeed, the sequencer error rate (sER) of SJ is also significantly (Two-sided Wilcox rank sum test P<0.01) higher than that of HAIB. The consistent significantly (Two-sided Wilcox rank sum test P<0.01) lower overall error rate of Q5 than that of Kapa is consistent with our previous findings 8. This data supports the value of measuring sER because lower sequencer error rate can result in lower overall error rate and measuring sER might help choosing best sequencers for deep sequencing applications.*

-An additional approach able to accurately measure the error-rates is Safe-SeqS. High-fidelity sequencing protocol, known as Safe-SeqS (https://academic.oup.com/bioinformatics/article/30/12/i329/392298) is able to correct errors in the NGS data by grouping the reads originated from the same barcode.

*[Response]: We agree that barcode-based approaches such as Safe-SeqS was designed to correct errors. However it is not designed to uncover sequencer error rates as presented in this work.*

*We added following in Discussion:*

*It should be noted that barcode-based sequencing methods, such as Safe-Seq and UMI methods (designed to suppress sequencing errors (both PCR errors and sequencer errors) through redundant sequencing followed by read collapsing within read families labeled by the same barcode) are an effective experimental error suppression method, but it cannot be used to specifically measure the sequencer errors because PCR errors and sequencer errors cannot be separated by barcode sequencing technology (Fig. 1a-b). On the other hand, because our method can provide accuracy information on sequencer, we believe an integrative approach of our method with barcode sequencing methods can result in a further improvement of overall sequencing accuracy, which will be our future study focus.*

--Availability of original (with errors) data and error-free data will allow to accurate estimate the error rates

*[Response]: The SRA ID of all samples are provided in Table S1a in the original submission.*

-Terminology needs to be clarified. Difference between platforms and sequencer needs to be clear defined Why samples with modified read names dropped?

*[Response]: In Introduction, we clarified this as “to make informed decisions about platform (e.g., HiSeq vs NovaSeq) and sequencer (i.e., actual instrument) choices for deep sequencing applications”.*

In section “Read name”, we stated that the tile/lane/surface/sequencer ID information are encoded in read names. Lack of such information from read names will render it impossible for our study (see Supplementary Note 2-3) which is why samples with modified read names were excluded from our analysis.

-Was all 3777 samples WGS samples? It would be interesting to compare the error rates between technologies (WES, WGS, RNA-Seq) potentially by sing the samples derived from the same individuals

*[Response]: This is a great idea. However, given this is a highly heterogeneous dataset (generally true for public datasets), this analysis is not possible. A specifically designed experiment is needed to test this hypothesis: the DNA source should be from a normal tissue from a healthy donor; the WGS/WES/RNA libraries better to be pooled and sequenced on the same sequencer/flow cell to minimize technical bias as we have demonstrated in this work. We do not currently have capacity for this experiment as part of this work.*

-There is a big discussion in the community on the value of the PHREAD scores? Were PHREAD scores used by the proposed method? Also what was the distribution of the PHREAD scores of bases determined as erroneous? Were there any erroneous bases which have very high PHREAD scores?

*[Response]: Thanks to this referee, our study in this work indicated that Phred score is not properly reflecting the base calling reliabilities. In our Algorithm section, we have stated that bases with Phred score lower than 30 were discarded. Therefore, all erroneous bases (i.e., mismatches between forward and reverse reads) detected by our method have very high Phred scores. This is why we believe our detected errors are truly sequencer errors.*

*We have added a section in Discussion to elaborate this: In addition to sER, our reported overall error rate (oER) of 10-100 pm (Fig. 1hs), which includes other error sources such as PCR errors, is 10- to 100-fold lower than generally reported accuracy of next generation sequencing methods of 1000 pm (10-3) 2. This reflects the successful “suppression” of poor-quality subset of data by our novel strategy. On the other hand, this raised an interesting perspective on the base call quality scores, known as Phred score. Traditional Phred scores were calculated based on sequencing traces of A/C/G/T bases by summarizing it to features such as peak spacing, uncalled/called ratio and peak resolution 23. The feature of called/uncalled ratio is a particularly interesting parameter, in that it measures the contrast between the true base and the other three non-bases of the corresponding position. Because noise is intrinsic to any scientific measure 2, signal strength of the uncalled bases is generally not 0. However, with the improvement of chemistry and physics, it is highly likely that the signal strength (i.e., noise level) of the non-bases will approach 0. Therefore, a non-0 pseudo number might have been used in the proprietary base call algorithms to prevent floating point overflow problem. For example, the current NovaSeq platforms only report Phred score up to 42, which correspond to P value of ~10-4. Because our data indicated that the calls at many bases have error rate close to 10-5 (at least in NovaSeq), we believe a re-evaluation of current Phred score calculation is warranted by the instrument manufacturer.*

-It would be beneficial to add discussion how proposed approach compares to the existing computational error correction methods (https://genomebiology.biomedcentral.com/articles/10.1186/s13059-020-01988-3)

*[Response]: Thanks so much for this suggestion. We performed the analysis and discovered that error correction methods do not provide much help, presumably because the overlap read structure was not accounted for by the DNA context-based error correction.*

*We added a section “Comparison of SequencErr with other error correction methods”: In addition to our error suppression method, which operates by identifying and filtering (i.e., suppression) unreliable reads, considerable efforts have been devoted to error correction methods, which operates by identifying DNA contexts (i.e., k-mer) that are error prone and followed by modifying (i.e., correction) corresponding readout. To benchmark with these methods, we focused on two error correction methods, Lighter (v1.1.2) 20 and Musket (v1.1) 21, that are considered to have top performance in a recent study by Mitchell et al. 22. We focused the analysis by using two representative samples (ERR3781298 and ERR3790800, Table S1e) sequenced using SJ and HAIB sequencers from previously published dataset 8 where the mutant and wildtype sites are well-defined (Table S3b). Here, the raw fastq files were ran through Lighter and Musket to correct errors, followed by standard pileup (Method). As can be seen in Fig. S11a-b, the overall sequencing error rate (oER) obtained by SequencErr far outperforms that of Lighter and Musket for all 12 possible nucleotide changes, although for C>T/G>A changes the difference is least dramatic. Interestingly, the overall error rate of Lighter and Musket does not appear to show improvement as compared to direct standard pileup. We next hypothesized that DNA sequence context-based modifications may lead to over-correction, which could be reflected in overlapping forward-reverse reads* which are *expected to have perfect matches. An increase in mismatch rate would indicate over-correction. Indeed, we observed ~10-fold increased forward-reverse mismatches in this dataset by Lighter or Musket than no correction (SequencErr measurement) (Fig. S11c-d). Taken together, we have demonstrated that our error suppression method outperforms error correction methods.*

*Fig S11. Comparison of SequencErr with error correction methods. Shown are overall error rate (oER) distribution of SequencErr (red), Lighter (green), Musket (blue), and raw data without error correction/suppression (No correction, purple), stratified by 12 possible nucleotide changes, for dataset generated by St. Jude (a) and HudsonAlpha (b). Also shown are sequencer error rate (sER) estimated by overlapping read pair method for data generated by St. Jude (c) and HudsonAlpha (d). In (c) and (d), sequencer error rate becomes higher (~10-fold) than no correction (SequencErr measurement), indicating over-correction.*

-Was any effect of read length observed. For example for similar technology was it more errors in sort reads compared to long?

*[Response]: We performed this analysis and found no significant correlation with read length.*

*We added a section “Effect of DNA sequencing features on sequencer error rate”: We next studied the effect of DNA sequence features on sequencer error rates, by focusing on 1) GC content; 2) read length; and 3) overall base quality, measured as percentage of bases with Phred score greater than 30 (Q30 Percentage). Because large number of samples are needed to draw robust conclusion, here we focused on 1,167 publicly available HiSeq datasets (Table S1b). As seen from Fig. S10, we did not detect significant correlation between sER score and GC content and read length features, though a marginally significant negative correlation with Q30 percentage is observed, indicating that excessive percentage of low quality bases can lead to unreliable sequencing output, even if after stringent quality filtering. This indicates that our SequencErr metric being highly robust in measuring sequencer reliability in a wide range of parameter settings.*

*Fig S10. Effect of DNA sequencing features on sequencer error rate. Shown are the effect of GC content (a), read length (b), and overall Q30 percentage (c). A linear model was fit for each feature, with R-squared and corresponding P value indicated.*

*This result is consistent with our detailed benchmarking with FastQC in section “Comparison of SequencErr with FastQC”, where sequencing cycles (another measure of read length): Because SequencErr is designed to understand sequencing quality, which is conceptually similar to FastQC 19—a frequently used quality control tool for sequencing datasets—we also benchmarked our method against FastQC. However, these two methods are fundamentally different in their design principle. FastQC operates on Phred scores (commonly known as Q30 percentage, which measures percentage of bases with Phred score above 30), but not the actual sequencing readout. On the other hand, SequencErr operates on actual sequencing readouts, although certain quality filters including Phred score distribution is applied to remove poor quality data. We used the previously published dataset 8 for this comparison. As seen in Fig. S9a, the FastQC evaluation shows that the Q30 percentage is >98% across all sequencing cycles, which would be considered good quality. However, by using our formula 1 at sequencing cycle level, we observed a stable error rate of ~11 per million across the sequencing cycles (Fig. S9b). Strikingly, we were able to observe the effect of problematic tiles on the sequencing cycles (Fig. S9b,c,f), which cannot be observed by reviewing FastQC output file (Fig. S9a,e).*

*Fig S9. Benchmarking SequencErr with FastQC. (a). Phred score distribution of read 1 and read 2 generated by FastQC from a representative sample (NCBI SRA ERR3781298). This dataset is considered high quality by FastQC analysis. Thick blue line indicates the median Phred score at given position (all above Phred score 36), and a thick dashed black line indicates the lower bound. (b) SequencErr analysis of the same dataset. Error rate (sER) of ~11 per million (thick red line) is observed along with sequencing cycles 30-100, without obvious difference between read 1 and read 2. (c) Effect of outlier tiles. By including the outlier tile (# 2201), cycles 77 (sER=210) and 92 (sER=230) in read 2 demonstrate elevated error rate. (d) The first 30 base pairs are not well covered (<0.1 million depth) due to limited read 1 and read 2 overlap, which depends on study design of insert size and actual read length. This explains the outlier error rate profile (mostly 0, a high number in position 30) from position 1 to position 30 in panels b and c. We also show tile-level quality based on FastQC output (e) and SequencErr output (f). In FastQC figure (panel e), the average Q score data (in tile × read position format) were position-wise standardized by using formula xt,i-µi, where µi is the average Q score of all tiles at read position i, and xt,i is the average Q score at read position i of tile t. In panel e, red arrows indicate poor Q score of tile # 2201 at read positions 76-77 (score: -3.3) and 92-93 (score: -3.59), which is consistent with our SequencErr results (panel c).*

-Did the authors observed a decrease in error rate over time for a given sequencing machine type.

*[Response]: This is excellent point. In our Fig. 2 we have shown that error rate appears to be stable for multiple runs (that happens over time) for a given sequencer. However, we currently do not have enough data to study the performance of a given platform (e.g., HiSeq/NovaSeq) over time. On the other hand, we have put our pipeline to SJ Cloud (mentioned in this submission) to enable analyzing large number of datasets in future.*

*We added to section “Comparison of sequencer error rates between platforms”: On the other hand, flow cell-level sER appears to be highly stable across runs within the non-outlier sequencers (Figs. 2b, S2), indicating that a successful initial sequencing experiment may ensure generation of high-quality data across many flow cells.*

-Did the author consider non human organisms? Is it expected to have a different error profile across various organisms?

*[Response]: We added an analysis on a recent SARS-CoV-2 dataset as non-human dataset and discovered that the error rate is comparable to those of human dataset. However, due to the numerous non-human species, we were not able to provide a comprehensive analysis in this dimension.*

*We added a new section “Application of SequencErr on non-human dataset”: Although we have demonstrated SequencErr method can be used to measure sequencer fidelity in above studies, it remains unknown whether it can be applied to non-human datasets. For this purpose, we applied SequencErr on a recently published Severe acute respiratory syndrome coronavirus 2 (SARS-CoV-2) dataset (NCBI SRA BioProject PRJNA625551) to study the error rate of corresponding sequencer. As can be seen in Fig. S12, the involving flowcells/sequencer demonstrated a comparable sER with those observed in human datasets (red dashed line). We therefore conclude that our method can also be applied to non-human datasets, although we believe that a more extensive study on many different non-human datasets could significantly strengthen this conclusion, which is beyond the scope of this work.*

Fig S12. Application of SequencErr on non-human dataset (SARS-CoV-2). Tile-level boxplot of sequencer error rate (y-axis) from 6 flowcells (x-axis) appears to be comparable to that (red dashed line) of human genome sequencing.

-In section "Public samples acquisition" It is unclear what the authors mean by "For reproducibility". The authors use the public data not for reproducibility. But because this data is available -Are samples names of 3777 samples are available?

*[Response]: We have removed “For reproducibility” to avoid confusion. The SRA ID of all samples are provided in Table S1a in the submission.*

-To allow a non-expert audience to easily understand the concepts of lanes, tiles, and surfaces, I would recommend including a figure to illustrate flow cells.

*[Response]: A cartoon has been added.*

*We added in Introduction: “We investigated error patterns associated with platforms, sequencers, flow cells, and tiles in flow cells (see Supplementary Note 1 for cartoon illustration) by using 3,777 datasets from 75 research institutions in 18 countries (Table S1a; Methods)”*

*Supplementary Note 1. Illustration of flowcell architecture.*

*Illustrated are top/bottom surface, lane, swath, and tiles, and how such information are encoded in read names.*

**Second round of review**

**Reviewer 1**

This manuscript, "SequencErr: Measuring and suppressing sequencer errors in next-generation sequencing" describes an approach which combines filtering based on read quality, mapping quality, and other data features (previously described as CleanDeepSeq) in order to suppress sequencing errors, and extends this approach by comparing mismatches between paired end reads to directly measure error rates due to sequencing (on the subset of pre-cleaned data). The authors compare sequencing error rates among a number of sequencing platforms using a large data set from SRA. In addition, they show that additional error suppression can be achieved by identifying and removing data from outlier tiles with elevated error rates.

The additional text, analysis, and clarifications have strengthened the resubmitted manuscript and based on this additional information, I have reassessed several of the objections raised in my previous review.

For instance, a previous comment (that error rates appeared underestimated) was based on my misunderstanding of what was shown in the figure. Thanks to the authors for clarifying in their comments and revisions to the text that these plots were not based off of raw sequencing data, but rather data in which error suppression strategies had already been applied. They may want to further emphasize that these sequencer and flow cell surface differences persist even after initial error suppression.

The comparison to other computational error suppression or correction algorithms also helps to contextualize the analysis presented in the paper and the utility of this approach. The authors also improved the paper by examining the effect of other factors such as read length, GC content, and reported Q-score on their analysis and compared the analysis with the SequencErr tool to the commonly used FastQC tool.

Finally, text was added to help clarify how this approach of computational error suppression on the basis of sequencer errors is differentiated from approaches such as UMI-based consensus correction which aims to lower error rates due to both library prep and sequencing issues.

I think there are a few outstanding issues to be addressed, but I would support publication if these can be addressed.

Major comments:

1) The ability to accurately assess discordance between the overlapping reads will be dependent on the ability to accurately identify regions of overlap. It looks like the authors are using overlapping alignements to a reference genome to identify overlap (as opposed to stitching or merging the reads). If this is the case, they should indicate this limitation of the approach/tool by revising the text (that it can only be applied to reference-based analysis).

2) Ideally, it would be nice to see the raw error rates (based on overlappinng read sections without the extensive upstream quality filtering) along with the suppressed error rates provided in the current figures. This might be complicated due to the alignment-based strategy (for instance, failing to do upstream quality filtering in order to measure the "true" sequencer error rate, may degrade the quality of the alignment and complicate the identification of bona fide overlapping regions). However, this question could be tackled using an amplicon data set where the amount of overlap and the expected read alignment is known (for instance, adding a "raw error rate" panel to figure 1h). This would help to quantify the magnitude of improvement enabled by this approach.

3) In order to comply with Genome Biology's open source and open data policies, both the source code and the SequencErr tool should be made available in an easily accessable location. Currently, it looks like the app is behind a password protected login which will limit the use and impact of the tool.

4) In the "Comparison of SequencErr with FastQC" section there are a few statements that are incorrect or need clarification. First, on line 49-51, they say "FastQC operates on Phred scores (commonly known as Q30 percentage)". This is a mis-statement about what Phred scores are. Phred scores are defined as ten times the log of the sequencing error rate (so 1 error in 1000 is log(1000) = 3*10 = Q30). FastQC takes in and plots the full range of Q-scores across cycles (though percent above Q30 is one metric that is reported). Also, in this section, it is not clear what is meant by the statement that "On the other hand, SequencErr operates on actual sequencing readouts". As far as I can tell, SequencErr makes use of an alignment file (like a sam or bam file), which is a more derived data type than a raw fastq file. This should be clarified to state the input for each program. I think that what is probably meant by this sentence is that in contrast to FastQC, SequencErr compares the actual sequence identity of aligned forward and reverse reads, but this needs to be stated more clearly (as the current sentence implies that SequencErr is somehow using raw data off the sequencer upstream of what FastQC uses, such as the raw imaging data, which is not the case).

5) Please note in the figure legends for Figures 2 and 3 (and any supplemental plots that show error rate measurements) that the rates shown are data which have been subjected upstream to error suppression (and are not raw error rates)

Minor comments:

1) In the text added to the section "Comparison of overall error rate (oER) and sequencer error rate (sER) measures", the authors state at the very end of this section that sER measurements may help choose the best sequencers for deep sequencing applications. This sentence is a bit ambiguous (do they mean type of sequencer - i.e. NextSeq vs. NovaSeq, or specific sequencer - NextSeq 1 vs. NextSeq 2). It would help to clarify this. In addition, loading conditions (cluster density, PhiX, etc.) can play a big role in quality. So they may want to add choosing the best sequencing "conditions" to this sentence as well.

2) Conclusions section - discussion of Phred scores lines 43-52 - discussion of signal strength, floating point numbers, etc. seems speculative and not relevant to the paper. On line 42, a may also want to add a comment about how the binning strategy employed to enable better data compression on the NovaSeq (in addition to the maximum Q-score of 42) makes Q-score estimates on this platform less accurate.

Text issues:

1) I would suggest that the title be changed to: "SequencErr: Measuring and suppressing sequencer errors in next-generation sequencing data"

2) Abstract Line 24: Should read "correspondance between overlapping regions"

3) Abstract Line 40: The last sentence of the abstract has some grammatical errors. I suggest correcting to "We demonstrate the SequencErr can reveal novel insights relative to the popular quality control method FastQC..."

4) Abstract Line 40: Change to "achieves a 10-fold lower".

5) Abstract Line 49: Change to "incurred in sequencing" or "incurred on DNA sequencers"

6) In the section "Comparison of overall error rate (oER) and sequencer error rate (sER) measures", line 29 should read: "in Fig. 1a-c, the overall error rate (oER..."

7) In the "Application of SequencErr on non-human dataset" section: line 35. changed "the involving" to "the involved" or re-word.

8) In the text added to the last page of the discussion (line 38) change to "because our method can provide accuracy information on the sequencer".

**Reviewer 2**

I am happy how comments were addressed

**Authors Response**

**Point-by-point responses to the reviewers’ comments:**

Reviewer reports:

Reviewer #1: This manuscript, "SequencErr: Measuring and suppressing sequencer errors in next-generation sequencing" describes an approach which combines filtering based on read quality, mapping quality, and other data features (previously described as CleanDeepSeq) in order to suppress sequencing errors, and extends this approach by comparing mismatches between paired end reads to directly measure error rates due to sequencing (on the subset of pre-cleaned data). The authors compare sequencing error rates among a number of sequencing platforms using a large data set from SRA. In addition, they show that additional error suppression can be achieved by identifying and removing data from outlier tiles with elevated error rates.

The additional text, analysis, and clarifications have strengthened the resubmitted manuscript and based on this additional information, I have reassessed several of the objections raised in my previous review.

For instance, a previous comment (that error rates appeared underestimated) was based on my misunderstanding of what was shown in the figure. Thanks to the authors for clarifying in their comments and revisions to the text that these plots were not based off of raw sequencing data, but rather data in which error suppression strategies had already been applied. They may want to further emphasize that these sequencer and flow cell surface differences persist even after initial error suppression.

*[Response]: We have updated the Discussion section as follows:*

*“Within non-outlier sequencers, the error rates of different flow cells appear to be rather stable, although there is 2.7% chance of observing outlier flow cells, indicating the need to continuously monitor the accuracy of sequencers. At flow cell level, we found that it is common to observe a small fraction (usually less than 10% in NextSeq and NovaSeq, 68% in HiSeq bottom surface) of tiles with exceedingly high error rates. Overall, >90% of HiSeq and NovaSeq flow cells have at least one outlier tiles. We also discovered that bottom surfaces of flow cells tend to have higher sequencer error rates. The bottom surface problem tends to be alleviated in NextSeq and NovaSeq compared to HiSeq sequencers. This data also indicates that sequencer, flow cell, and flow cell surface differences (in terms of error rate) persist even after initial error suppression.”*

The comparison to other computational error suppression or correction algorithms also helps to contextualize the analysis presented in the paper and the utility of this approach. The authors also improved the paper by examining the effect of other factors such as read length, GC content, and reported Q-score on their analysis and compared the analysis with the SequencErr tool to the commonly used FastQC tool.

Finally, text was added to help clarify how this approach of computational error suppression on the basis of sequencer errors is differentiated from approaches such as UMI-based consensus correction which aims to lower error rates due to both library prep and sequencing issues.

*We greatly appreciate the referee’s recognition of our study. Thank you very much!*

I think there are a few outstanding issues to be addressed, but I would support publication if these can be addressed.

Major comments:

1) The ability to accurately assess discordance between the overlapping reads will be dependent on the ability to accurately identify regions of overlap. It looks like the authors are using overlapping alignements to a reference genome to identify overlap (as opposed to stitching or merging the reads). If this is the case, they should indicate this limitation of the approach/tool by revising the text (that it can only be applied to reference-based analysis).

*[Response]: We added a statement in section “Discussion” to indicate this limitation:*

*“…indicates that the small fraction of short inserts in conventional WGS libraries may provide enough information to assess sequencer error rate. Moreover, reference mapping is required for our approach to determine the overlapping regions between forward and reverse reads, which renders it challenging to apply our method to sequencing data from a species without a reference genome available.”*

2) Ideally, it would be nice to see the raw error rates (based on overlappinng read sections without the extensive upstream quality filtering) along with the suppressed error rates provided in the current figures. This might be complicated due to the alignment-based strategy (for instance, failing to do upstream quality filtering in order to measure the "true" sequencer error rate, may degrade the quality of the alignment and complicate the identification of bona fide overlapping regions). However, this question could be tackled using an amplicon data set where the amount of overlap and the expected read alignment is known (for instance, adding a "raw error rate" panel to figure 1h). This would help to quantify the magnitude of improvement enabled by this approach.

*[Response]: Thanks for this nice suggestion, our analysis demonstrated a dramatic effect of our computational error suppression. We added a new panel in Fig. 1h as follows:*

*We added a new paragraph for this new data: “To understand the effect of computational error suppression in sequencer error rate (sER), we performed a similar analysis as above except without computational error suppression (i.e., no quality filtering on mapping quality and Phred scores). As a result (Fig. 1h), the sER is now close to 1000 pm (10-3), which is consistent with previous reports 2,6. This result reinforced our previous observation that computational error suppression can lead to 10- to 100- fold error rate reduction 8. With this observation, we will apply computational error suppression to calculate sER hereafter unless otherwise stated.”*

3) In order to comply with Genome Biology's open source and open data policies, both the source code and the SequencErr tool should be made available in an easily accessable location. Currently, it looks like the app is behind a password protected login which will limit the use and impact of the tool.

*[Response]: We have deposited the source code to Zenodo (http://doi.org/10.5281/zenodo.4313906 and https://zenodo.org/record/4312180).*

*The Cloud tool enables user to test thousands of samples without the need of downloading—which is very challenging both in terms of storage and time. Academic users can create account in the Cloud and directly run our tool without restrictions. The username/password we provided is a temporary Cloud account to keep the reviewers anonymous without the need to create an account to test our tools, therefore we did not limit the use of the tool.*

*To avoid this confusion, we have added a new section “SequencErr on St. Jude Cloud” in Method:*

*“An end-to-end pipeline deployable through a graphical point-and-click interface is available on St. Jude Cloud (https://platform.stjude.cloud/workflows/sequencerr). Academic users can create an account in St. Jude Cloud and run this pipeline without restrictions.”*

4) In the "Comparison of SequencErr with FastQC" section there are a few statements that are incorrect or need clarification. First, on line 49-51, they say "FastQC operates on Phred scores (commonly known as Q30 percentage)". This is a mis-statement about what Phred scores are. Phred scores are defined as ten times the log of the sequencing error rate (so 1 error in 1000 is log(1000) = 3*10 = Q30). FastQC takes in and plots the full range of Q-scores across cycles (though percent above Q30 is one metric that is reported). Also, in this section, it is not clear what is meant by the statement that "On the other hand, SequencErr operates on actual sequencing readouts". As far as I can tell, SequencErr makes use of an alignment file (like a sam or bam file), which is a more derived data type than a raw fastq file. This should be clarified to state the input for each program. I think that what is probably meant by this sentence is that in contrast to FastQC, SequencErr compares the actual sequence identity of aligned forward and reverse reads, but this needs to be stated more clearly (as the current sentence implies that SequencErr is somehow using raw data off the sequencer upstream of what FastQC uses, such as the raw imaging data, which is not the case).

*[Response]: Thank you so much for nice suggestion! We have rephrased the statements as follows:*

*“FastQC operates on Phred scores of sequenced bases (regardless of the actual sequence identity) and measures a sample-level quality metric as the percent of bases with the Phred score above a given threshold, e.g., Q30 percentage. In contrast to FastQC, SequencErr compares the actual sequence identity of aligned forward and reverse reads, although quality filters including Phred score distribution are applied to remove poor quality data (see Methods).”*

5) Please note in the figure legends for Figures 2 and 3 (and any supplemental plots that show error rate measurements) that the rates shown are data which have been subjected upstream to error suppression (and are not raw error rates)

*[Response]: Excellent points! We added below statement to clarify this:*

*“…This result reinforced our previous observation that computational error suppression can lead to 10- to 100- fold error rate reduction 8. With this observation, we will apply computational error suppression to calculate sER hereafter unless otherwise stated.”*

Minor comments:

1) In the text added to the section "Comparison of overall error rate (oER) and sequencer error rate (sER) measures", the authors state at the very end of this section that sER measurements may help choose the best sequencers for deep sequencing applications. This sentence is a bit ambiguous (do they mean type of sequencer - i.e. NextSeq vs. NovaSeq, or specific sequencer - NextSeq 1 vs. NextSeq 2). It would help to clarify this. In addition, loading conditions (cluster density, PhiX, etc.) can play a big role in quality. So they may want to add choosing the best sequencing "conditions" to this sentence as well.

*[Response]: We added below statement to clarify this:*

*“…This data supports the value of measuring sER because lower sequencer error rate can result in lower overall error rate and measuring sER might help choosing the best sequencers for deep sequencing applications. For example, our data indicated that NovaSeq and NextSeq are on average preferred over HiSeq sequencers in terms of error rates. Because different sequencers can have dramatically different error rates (such as the two NovaSeq sequencers studied here), specific sequencers with lower error rates are preferred.”*

*Regarding the sequencing conditions, we added below statement in section “Discussion” to clarify:*

*“In this work, we were not able to study the effect of sequencing conditions such as cluster density and PhiX spike-in because such parameters are not reported in publicly available datasets. Future studies on these parameters are warranted.”*

2) Conclusions section - discussion of Phred scores lines 43-52 - discussion of signal strength, floating point numbers, etc. seems speculative and not relevant to the paper. On line 42, a may also want to add a comment about how the binning strategy employed to enable better data compression on the NovaSeq (in addition to the maximum Q-score of 42) makes Q-score estimates on this platform less accurate.

*[Response]: Thank you for the nice suggestion! We removed the statements in lines 43-52 (strikethrough below), and added a comment on the binning strategy:*

*“On the other hand, this raised an interesting perspective on the base call quality scores, known as Phred score. Traditional Phred scores were calculated based on sequencing traces of A/C/G/T bases by summarizing it to features such as peak spacing, uncalled/called ratio, and peak resolution 23. The feature of called/uncalled ratio is a particularly interesting parameter, in that it measures the contrast between the true base and the other three non-bases of the corresponding position. Because noise is intrinsic to any scientific measure 2, the signal strength of the uncalled bases is generally not 0. However, with the improvement of chemistry and physics, it is highly likely that the signal strength (i.e., noise level) of the non-bases will approach 0. Therefore, a non-0 pseudo number might have been used in the proprietary base call algorithm to prevent floating-point overflow problem. For example, the current NovaSeq platforms only report Phred to score up to 42, which corresponds to P-value of ~10-4. Because our data indicated that the calls at many bases have an error rate close to 10-5 (at least in NovaSeq), we believe a re-evaluation of the current Phred score calculation is warranted by the instrument manufacturer. For example, it would be interesting to determine if the Phred score binning strategy that was intended for better data compression in NovaSeq data may lead to less accurate Phred score estimation.”*

Text issues:

*[Response]: we really appreciate the referee’s great effort in improving our presentation. We have requested scientific editing and highlighted this in section “Acknowledgments”:*

*“We thank the two anonymous reviewers for their suggestions to improve the presentation. The authors are grateful to the scientific editing support by Makeda Porter-Carr.”*

1) I would suggest that the title be changed to: "SequencErr: Measuring and suppressing sequencer errors in next-generation sequencing data"

*[Response]: Updated as:*

*“SequencErr: measuring and suppressing sequencer errors in next-generation sequencing data”*

2) Abstract Line 24: Should read "correspondance between overlapping regions"

*[Response]: Updated as:*

*“by measuring base correspondence between overlapping regions in forward and reverse reads”*

3) Abstract Line 40: The last sentence of the abstract has some grammatical errors. I suggest correcting to "We demonstrate the SequencErr can reveal novel insights relative to the popular quality control method FastQC..."

*[Response]: Updated as:*

*“We demonstrate that SequencErr can reveal novel insights relative to popular quality control method FastQC…”*

4) Abstract Line 40: Change to "achieves a 10-fold lower".

*[Response]: Updated as:*

*“achieves a 10-fold lower”*

5) Abstract Line 49: Change to "incurred in sequencing" or "incurred on DNA sequencers"

*[Response]: Updated as:*

*“errors incurred on DNA sequencers”*

6) In the section "Comparison of overall error rate (oER) and sequencer error rate (sER) measures", line 29 should read: "in Fig. 1a-c, the overall error rate (oER..."

*[Response]: Updated as:*

*“in Fig. 1a-c, the overall error rate (oER…”*

7) In the "Application of SequencErr on non-human dataset" section: line 35. changed "the involving" to "the involved" or re-word.

*[Response]: Updated as:*

*“the involved flowcells/sequencer…”*

8) In the text added to the last page of the discussion (line 38) change to "because our method can provide accuracy information on the sequencer".

*[Response]: Updated as:*

*“because our method can provide accuracy information on the sequencer”*
